# An Injectable Oxygen Release System to Augment Cell Survival and Promote Cardiac Repair Following Myocardial Infarction

**DOI:** 10.1038/s41598-018-19906-w

**Published:** 2018-01-22

**Authors:** Zhaobo Fan, Zhaobin Xu, Hong Niu, Ning Gao, Ya Guan, Chao Li, Yu Dang, Xiaoyu Cui, Xuanyou Liu Liu, Yunyan Duan, Haichang Li, Xinyu Zhou, Pei-Hui Lin, Jianjie Ma, Jianjun Guan

**Affiliations:** 1 Department of Materials Science and Engineering, The Ohio State University, Columbus, OH 43210 USA; 2 Davis Heart and Lung Research Institute, The Ohio State University, Columbus, OH 43210 USA; 3 Department of Surgery, The Ohio State University, Columbus, OH 43210 USA

## Abstract

Oxygen deficiency after myocardial infarction (MI) leads to massive cardiac cell death. Protection of cardiac cells and promotion of cardiac repair are key therapeutic goals. These goals may be achieved by re-introducing oxygen into the infarcted area. Yet current systemic oxygen delivery approaches cannot efficiently diffuse oxygen into the infarcted area that has extremely low blood flow. In this work, we developed a new oxygen delivery system that can be delivered specifically to the infarcted tissue, and continuously release oxygen to protect the cardiac cells. The system was based on a thermosensitive, injectable and fast gelation hydrogel, and oxygen releasing microspheres. The fast gelation hydrogel was used to increase microsphere retention in the heart tissue. The system was able to continuously release oxygen for 4 weeks. The released oxygen significantly increased survival of cardiac cells under the hypoxic condition (1% O_2_) mimicking that of the infarcted hearts. It also reduced myofibroblast formation under hypoxic condition (1% O_2_). After implanting into infarcted hearts for 4 weeks, the released oxygen significantly augmented cell survival, decreased macrophage density, reduced collagen deposition and myofibroblast density, and stimulated tissue angiogenesis, leading to a significant increase in cardiac function.

## Introduction

MI causes massive death of cardiac cells including cardiomyocytes, cardiac fibroblasts and endothelial cells. Extremely low oxygen content in the infarcted area is a major cause of death^1–5^. MI also induces severe pathogenic inflammatory responses, scar formation, and cardiac function decrease^1–5^. Protection of cardiac cells and promotion of cardiac repair are key treatment goals^1–5^. These goals may be achieved by clinical reperfusion intervention that reintroduces oxygen into the infarcted heart. However, not all patients are eligible for this type of intervention^6,7^. Cell therapy has potential to use endogenous or exogenous cells for cardiac repair, yet cell survival is inferior in the low oxygen condition of the damaged hearts^8–16^. Biomaterial therapy with or without growth factors may aid myocardial repair by providing mechanical support to the heart tissue, and affecting tissue inflammation and angiogenesis^17–26^. However, the efficacy remains low due to their inability to provide oxygen to metabolic-demanding cardiac cells at early stage of tissue damage^15,16^. To address the critical need of oxygen to protect cardiac cells, direct supply of sufficient oxygen in the infarcted area while not provoking deleterious effects is necessary. However, this cannot be achieved by current oxygen therapy approaches.

Oxygen supplementation is a standard treatment for MI patients because it increases oxygen level in the blood of healthy tissues to avoid hypoxic damage caused by lower blood pumping ability after MI^27^. It may also augment oxygen level in the infarcted tissue to protect cardiac cells although this area has extremely low blood supply. As a result, cardiac function may improve^27–29^. Experiments using canine model have demonstrated that inhalation of 100% oxygen decreased infarct size and increased cardiac function (ejection fraction)^30^. Several clinical studies also showed similar effects when patients inhaled 100% oxygen^31–33^, yet some did not show any effect^34^.

Hyperbaric oxygen therapy uses 100% oxygen with high pressure (>1 atm). The purpose is to better increase blood oxygen level than traditional oxygen therapy^35–37^. Animal studies have shown that hyperbaric oxygen therapy increased cell survival in the infarcted hearts^36,37^. Some clinical studies demonstrated that hyperbaric oxygen therapy decreased end-systolic volume by 20% and increased cardiac output by 10%^38^. Yet other clinical studies did not have similar beneficial effects^39,40^. Intracoronary injection of arterial blood supersaturated with oxygen is also an approach to augment oxygen level in the infarcted area. Some clinical studies demonstrated that this approach can significantly improve cardiac function after 30 days for patients with large damaged area^41–43^. However, no positive effect was found in some other clinical studies^41–43^. Transfusion of oxygen carriers into blood after MI to increase blood oxygen level has been tested in animal models. The results demonstrated that infarct size was reduced and cardiomyocyte survival was increased^44–47^. However, clinical data on this approach is lacking.

Overall, current oxygen therapy for MI treatment is focused on systemic oxygen delivery, and the therapeutic efficacy is low. In addition, the results are inconsistent in clinical trials and preclinical studies^27–29^. This is because: (1) The infarcted area has extremely low blood flow, thus largely limiting oxygen in the blood to diffuse into the area^48^. The oxygen level may be too low to protect substantial number of cells; (2) systemic increase of blood oxygen level decreases coronary artery blood flow^49,50^. This directly decreases oxygen diffusion to the infarcted area; and (3) current approaches cannot increase oxygen level in the blood for prolonged period to continuously provide oxygen to the cardiac cells since the oxygen level decreases to the normal level shortly after the treatment. Prolonged inhalation of oxygen or injection of oxygen carriers into blood may lead to side effects on healthy tissues as the oxidative stress may be increased in these tissues causing cell death and tissue inflammation.

To address limitations of current oxygen therapy in order to largely augment its therapeutic efficacy, an approach that can efficiently deliver necessary level of oxygen to the infarcted area for a prolonged period while not causing side effects is critically necessary. In this work, we developed a new oxygen delivery system that can be delivered specifically to the infarcted tissue in order to avoid side effects on healthy tissues, and can gradually release sufficient oxygen to continuously oxygenate the metabolic-demanding cardiac cells. The effect of released oxygen on cardiac cell survival, myofibroblast formation, cardiac fibrosis, tissue inflammation, angiogenesis and cardiac function was studied.

## Results

### Fabrication of hydrogel-based oxygen release system and oxygen release kinetics

The oxygen release system was based on a thermal sensitive and fast gelation hydrogel, and oxygen release microspheres. The hydrogel polymer had NIPAAm/HEMA/AOLA ratio of 86.0/10.5/3.5 as determined from ^1^H-NMR spectrum. The 20% hydrogel solution was flowable at 4 °C, and can be readily injected through a 28-gauge needle. The hydrogel solution exhibited a thermal transition temperature of 26.7 °C. The gelation time was less than 7 s at 37 °C. After incubation in the DPBS for 4 weeks at 37 °C, the hydrogel showed 5.4% of weight loss.

**Figure 1:**
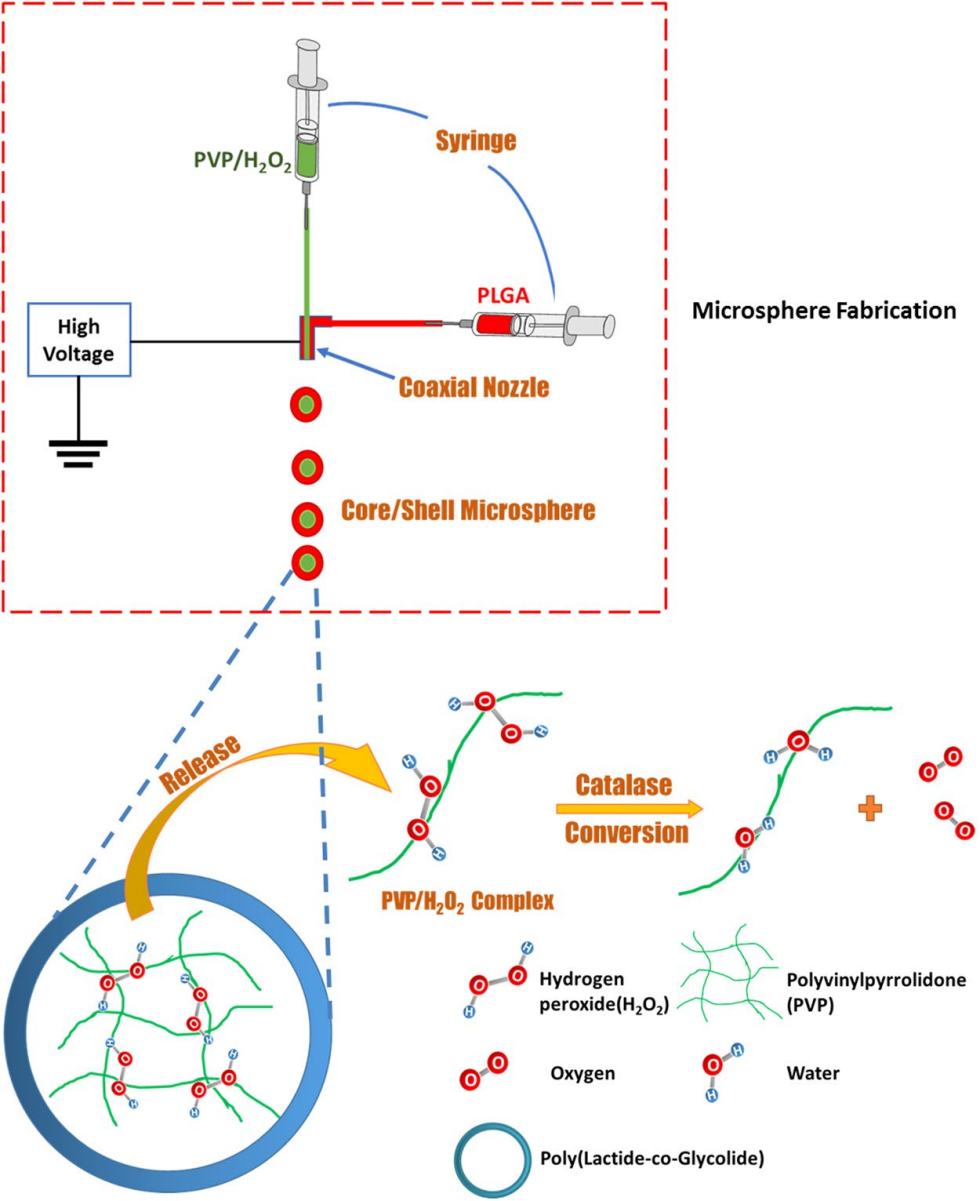
Oxygen release microsphere fabrication and oxygen release mechanism.

**Figure 2:**
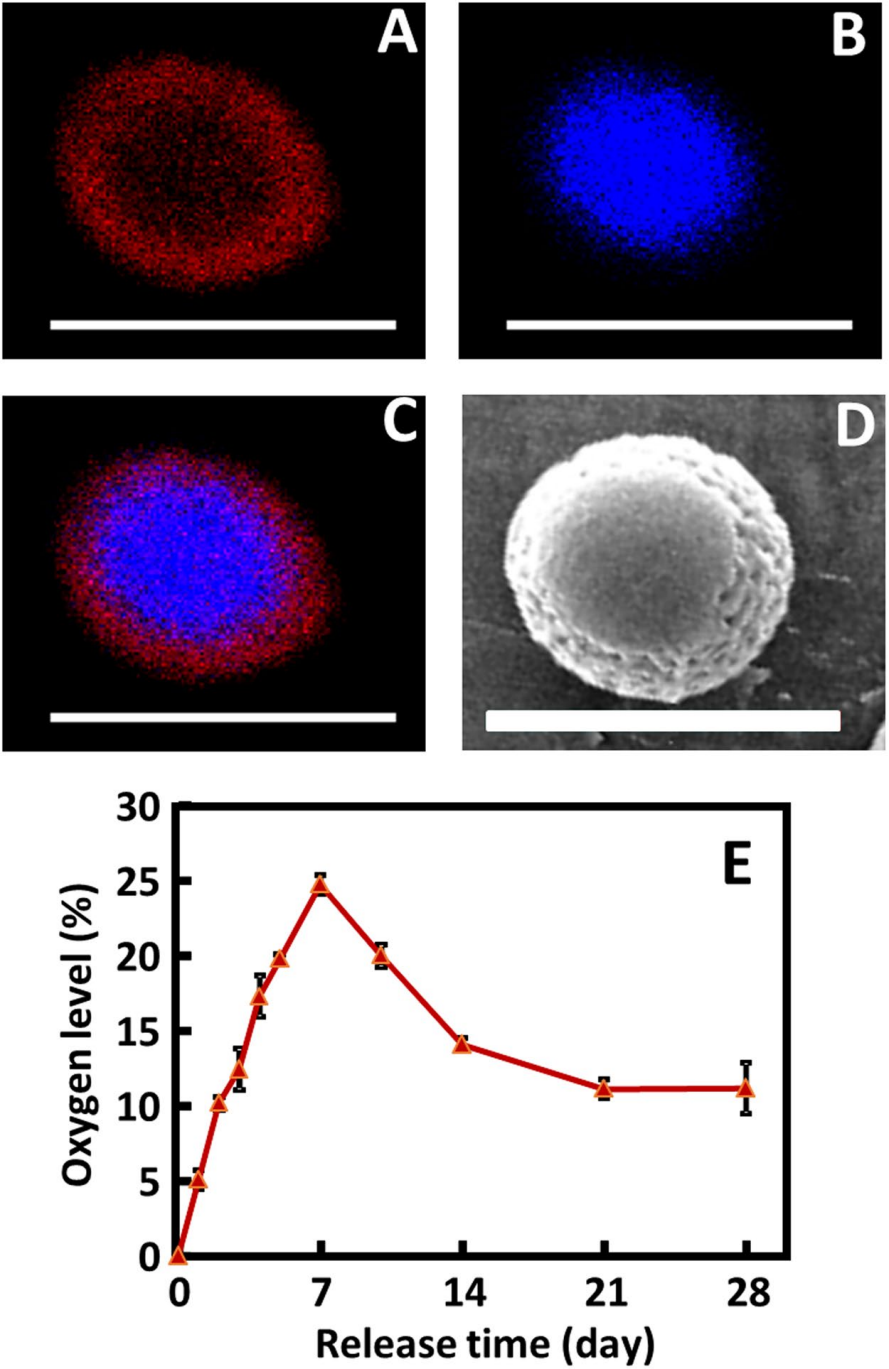
Core-shell structure of oxygen release microsphere and oxygen release kinetics. (**A**) PLGA shell; (**B**) PVP/H_2_O_2_ core; (**C**) combined core-shell structure. For imaging purpose, rhodamine and draq5 were added to the shell and core respectively before fabrication; (**D**) SEM image of microsphere; and (**E**) oxygen release kinetics during a 28-day release period. Scale bar = 5 µm.

The oxygen release microspheres were fabricated by electrospraying (Fig. 1), and had core-shell structure with PLGA as shell and PVP/H_2_O_2_ complex as core (Fig. 2). Oxygen was generated after the PVP/H_2_O_2_ complex released from microspheres was converted into molecular oxygen and water by catalase encapsulated in the hydrogel. The released oxygen was measured by using oxygen-sensitive luminophore Ru(Ph_2_phen_3_)Cl_2_ and oxygen-insensitive fluorophore rhodamine-B. The Ru(Ph_2_phen_3_)Cl_2_ has high quantum yield and oxygen quenching, and is thermal stable at physiological temperature^51,52^, while rhodamine-B allows for correction of typical errors, such as heterogeneous fluorophore concentration, bleaching, and fluctuations in excitation light intensity^53^. Figure. 2 demonstrates that the oxygen release system was able to continuously release oxygen in a 4-week testing period. The release showed a two phasic pattern. In the first 7 days, the oxygen level was gradually increased. After 1 day of release, the oxygen level was above 5%. The release was peaked at day 7 where oxygen level was 24.8%. After 7 days, the oxygen level was steadily decreased. By day 28, it was 11.2%.

### Effect of oxygen release on cell survival under hypoxic conditions

**Figure 3:**
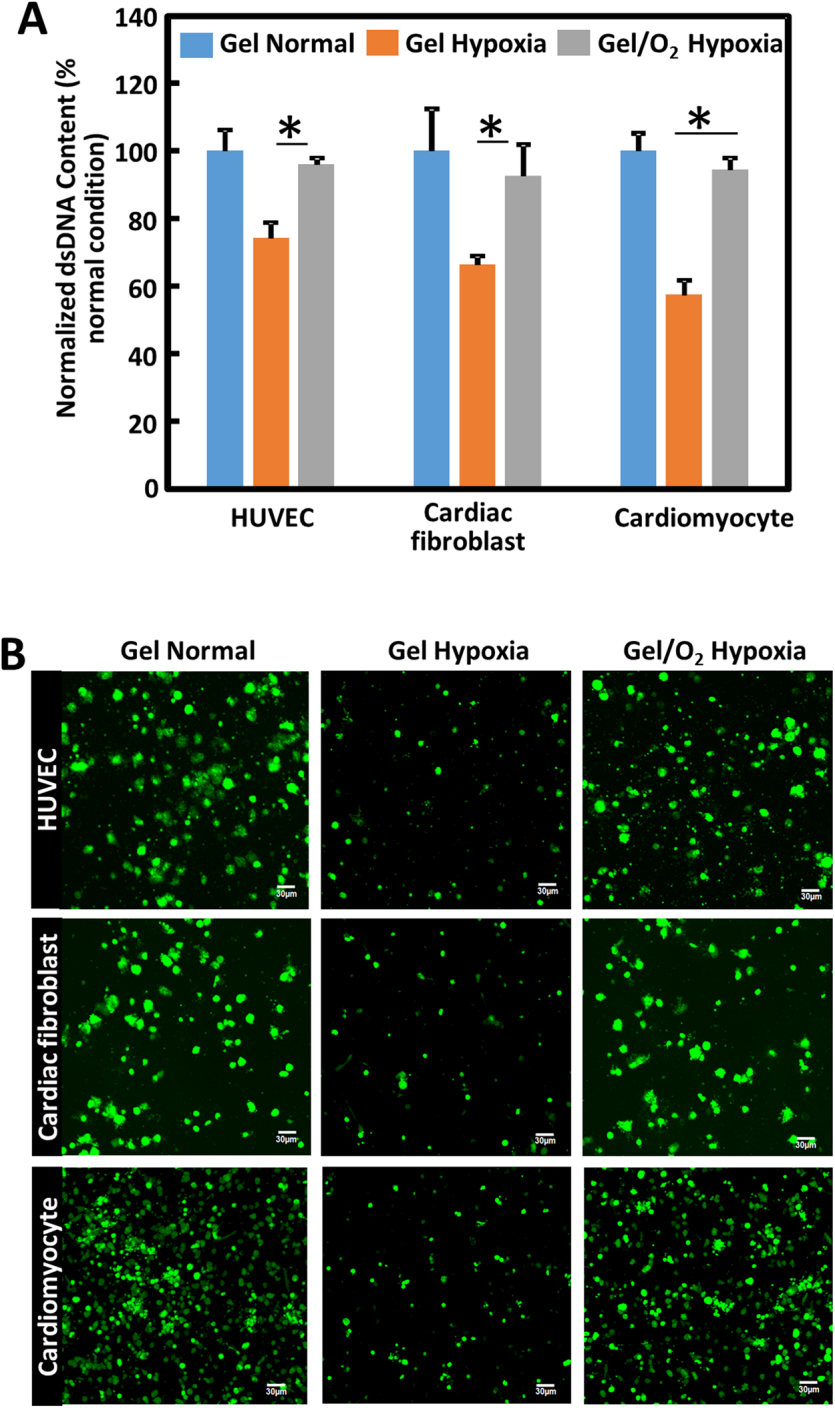
HUVECs, cardiac fibroblasts, and cardiomyocytes survival under normal and hypoxic conditions. (**A**) dsDNA content. Three cell types were seeded in 3D collagen gel, respectively. Hydrogel (Gel) was then injected into the collagen gel and cultured under normal (Gel Normal group) or 1% O_2_ (Gel Hypoxia group) conditions. Oxygen release system (Gel/O_2_) was also injected into the collagen gel but cultured under 1% O_2_ (Gel/O_2_ Hypoxia group). dsDNA was measured 2 days after injection. dsDNA under hypoxia was normalized to the collagen gel injected with Gel and cultured under normal condition; and (**B**) live cell images of HUVECs, cardiac fibroblasts, and cardiomyocytes in the 3D collagen gel. Cells were labeled with live cell tracker CMFDA before seeding. *p < 0.05. Scale bar = 30 µm.

To evaluate the survival of cardiomyocytes, endothelial cells and cardiac fibroblasts under hypoxic condition, each cell type was first seeded into 3D collagen gels. The oxygen release system was then injected into the collagen gels followed by culturing under 1% O_2_ condition (Gel/O_2_ Hypoxia group). Controls were collagen gels injected with hydrogel only and cultured under hypoxia (Gel Hypoxia group) or normal oxygen condition (Gel Normal group). Figure 3A shows that 1% O_2_ condition significantly decreased cell dsDNA content after 2 days of culture (p < 0.0001 for cardiomyocytes, and p < 0.01 for cardiac fibroblasts and HUVECs Fig 3A), demonstrating that this oxygen condition caused cell death. The injection of hydrogel only did not increase dsDNA content for 3 cell types (p > 0.05). In contrast, the injection of oxygen release system significantly augmented cell dsDNA content (p < 0.001 in cardiomyocytes, p < 0.01 in HUVECs, and p < 0.05 for cardiac fibroblasts), suggesting that the released oxygen increased cell survival under extremely low oxygen condition. The dsDNA content for each cell type was similar to that cultured under normal oxygen condition (Fig. 3A). To observe live cells, they were stained with a live cell tracker CMFDA before seeding into collagen gels. Consistent with dsDNA data, the cells cultured under 1% O_2_ condition with the injection of hydrogel showed dramatic lower cell density than those cultured under normal oxygen condition (Fig. 3B). The injection of oxygen release system increased cell density (Fig. 3B).

### Effect of oxygen release on cell ROS content

**Figure 4:**
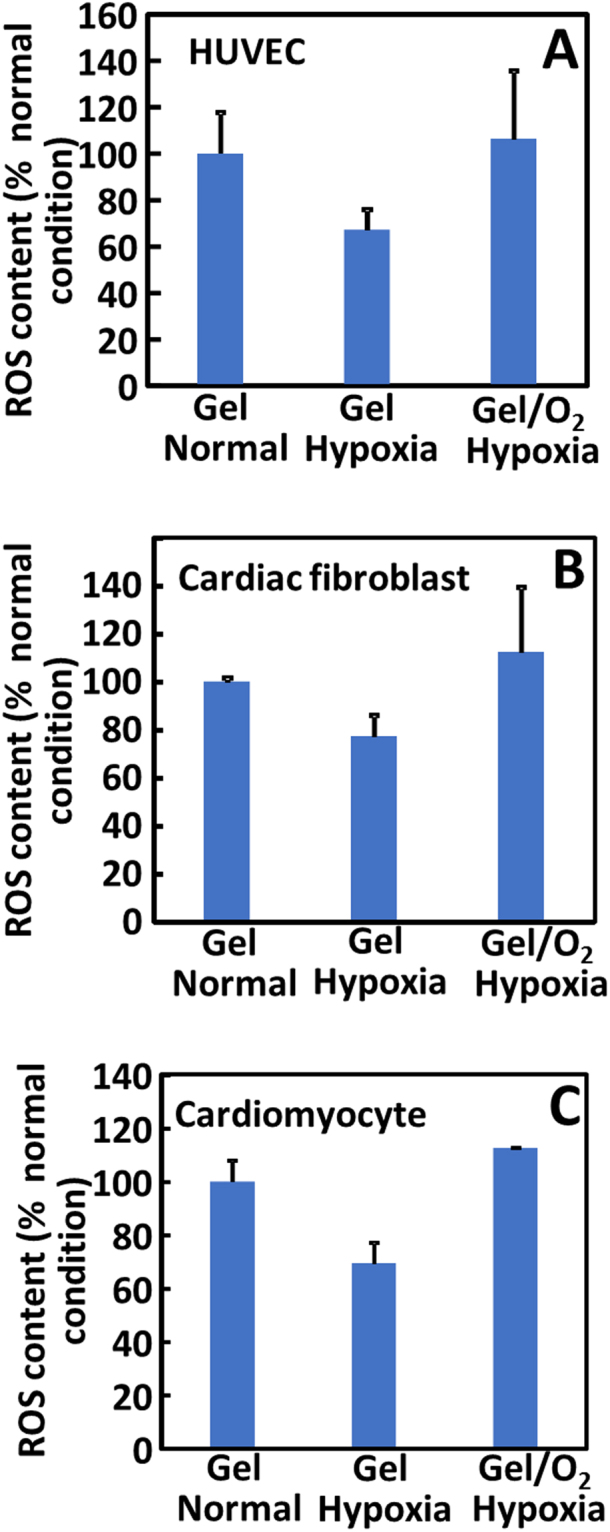
ROS content in HUVECs, cardiac fibroblasts, and cardiomyocytes seeded in collagen gels. The Gel and Gel/O_2_ groups were injected into the collagen gels respectively and cultured under normal condition or hypoxia. ROS content was measured 2 days after injection. (**A**) HUVECs; (**B**) Cardiac fibroblasts; and (**C**) cardiomyocytes. *p < 0.05.

To determine whether oxygen release affected cell ROS content, cardiomyocytes, HUVECs and cardiac fibroblasts were stained with a ROS sensitive dye CM-H_2_DCFDA before seeding into collagen gels. The relative ROS content was quantified. The 3 cell types showed different ROS contents under 3 different conditions (Fig. 4). At low oxygen condition (1% O_2_), the ROS contents were significantly decreased for each cell type (p < 0.0001) compared to the normal oxygen condition. Injection of the oxygen release system into the collagen gels significantly increased cell ROS content (p < 0.0001). However, the ROS content was similar to that of cells cultured under normal culture conditions (p > 0.1).

### Effect of oxygen release on myofibroblast formation under hypoxic condition

**Figure 5:**
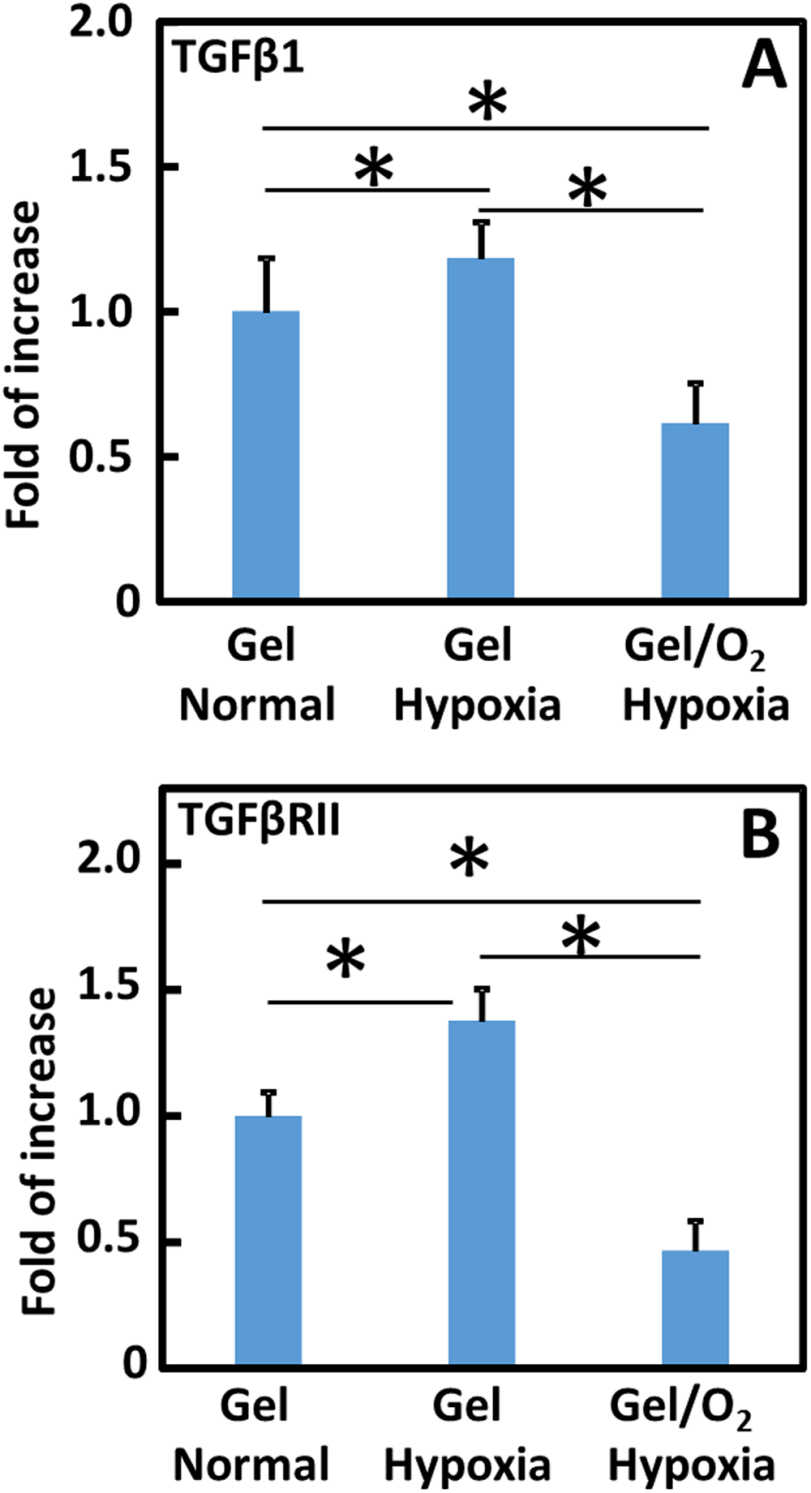
Gene expression of TGFβ1 (**A**) and TGFβRII (**B**) in cardiac fibroblasts when seeded in collagen gels. The Gel and Gel/O_2_ groups were injected into the collagen gels respectively and cultured under normal condition or hypoxia (1% O_2_). Gene expression was measured 2 days after injection. TGFβ1 (5 ng/mL) was added into the medium during culture. *p < 0.05.

To determine the effect of oxygen release on myofibroblast formation, the cardiac fibroblasts were seeded in the 3D collagen gel that can maintain fibroblast phenotype for at least 48 h^54^, and cultured in the medium supplemented with TGFβ1 under either normal oxygen or 1% O_2_ condition. After 2 days of culture, the expressions of TGFβ1 and TGFβRII at the mRNA level were significantly upregulated in the 1% O_2_ condition than in the normal oxygen condition (p < 0.05 for both expressions Fig. 5). This increases the possibility of TGFβ1 binding to TGFβRII, leading to the formation of myofibroblasts. Injection of oxygen release system into the collagen gel significantly downregulated the expressions of these genes (p < 0.05 Fig. 5).

**Figure 6:**
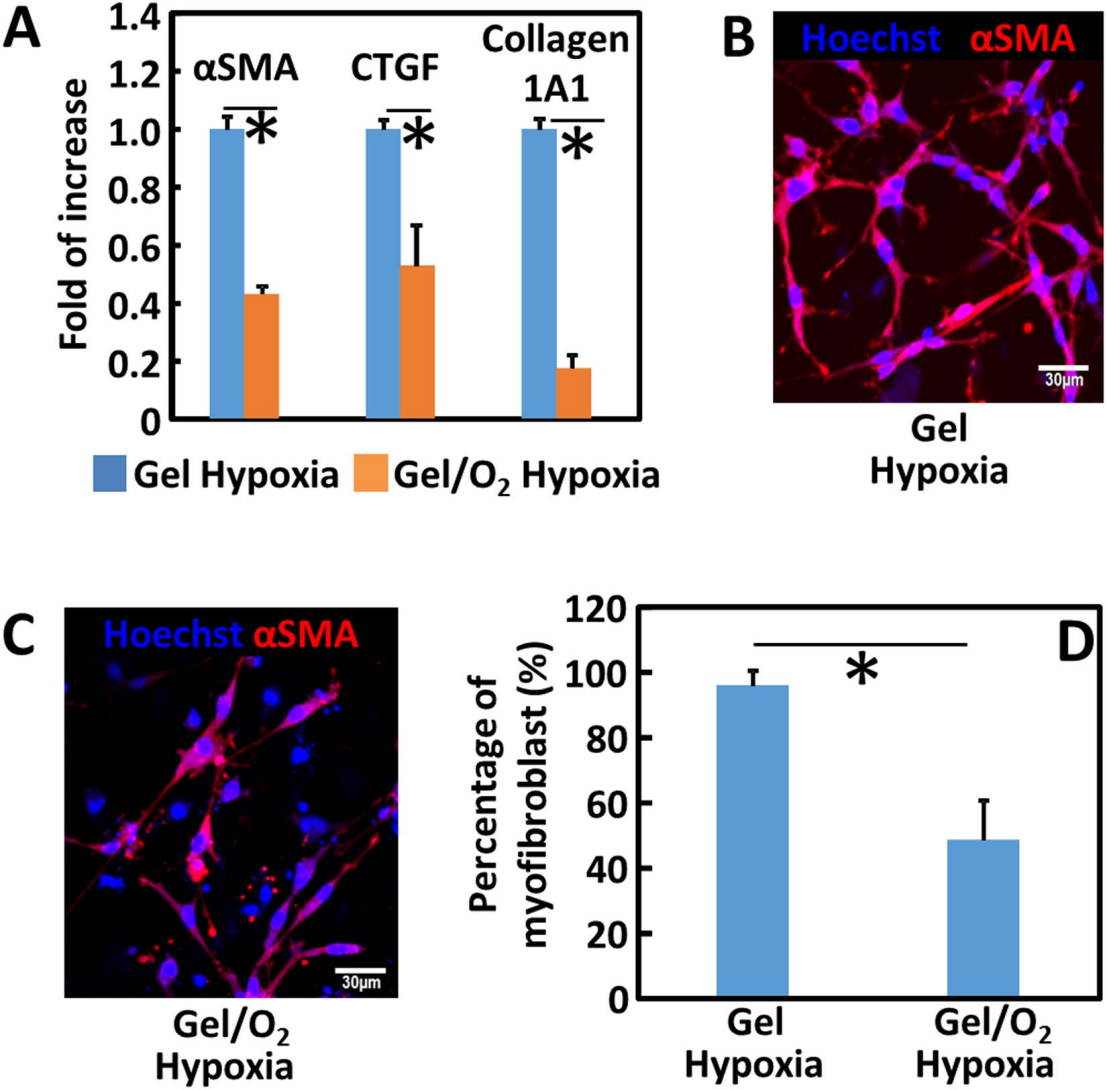
Cardiac fibroblasts differentiation into myofibroblasts. (**A**) Gene expressions of αSMA, CTGF and Collagen 1A1; (**B**,**C**) immunostaining of αSMA; and (**D**) percentage of αSMA+ myofibroblasts. Cardiac fibroblasts were seeded in collagen gels. The Gel and Gel/O_2_ groups were injected into the collagen gels respectively and cultured under hypoxia (1% O_2_). Gene and protein expressions were measured 2 days after injection. TGFβ1 (5 ng/mL) was added into the medium during culture. *p < 0.05. Scale bar = 30 µm.

To characterize cardiac fibroblast differentiation into myofibroblast, myofibroblast markers including αSMA, CTGF, and collagen1A1 were quantified at the mRNA level. The 3 markers were significantly downregulated after injection of oxygen release system into the collagen gel (Gel vs. Gel/O_2_ groups. p < 0.05 Fig. 6A). At the protein level, nearly all of the cells were αSMA positive myofibroblasts in the Gel group (Fig. 6B and D). The release of oxygen in the collagen gel (Gel/O_2_ group) significantly decreased the ratio of myofibroblasts to 49% (p < 0.05 Fig. 6C and D). The increase of myofibroblast ratio is not caused by gel as we found that cardiac fibroblasts cultured on the gel surface without adding TGFβ1 did not express αSMA (data not shown).

### Effect of oxygen release system on infarcted left ventricular wall thickness after MI

**Figure 7:**
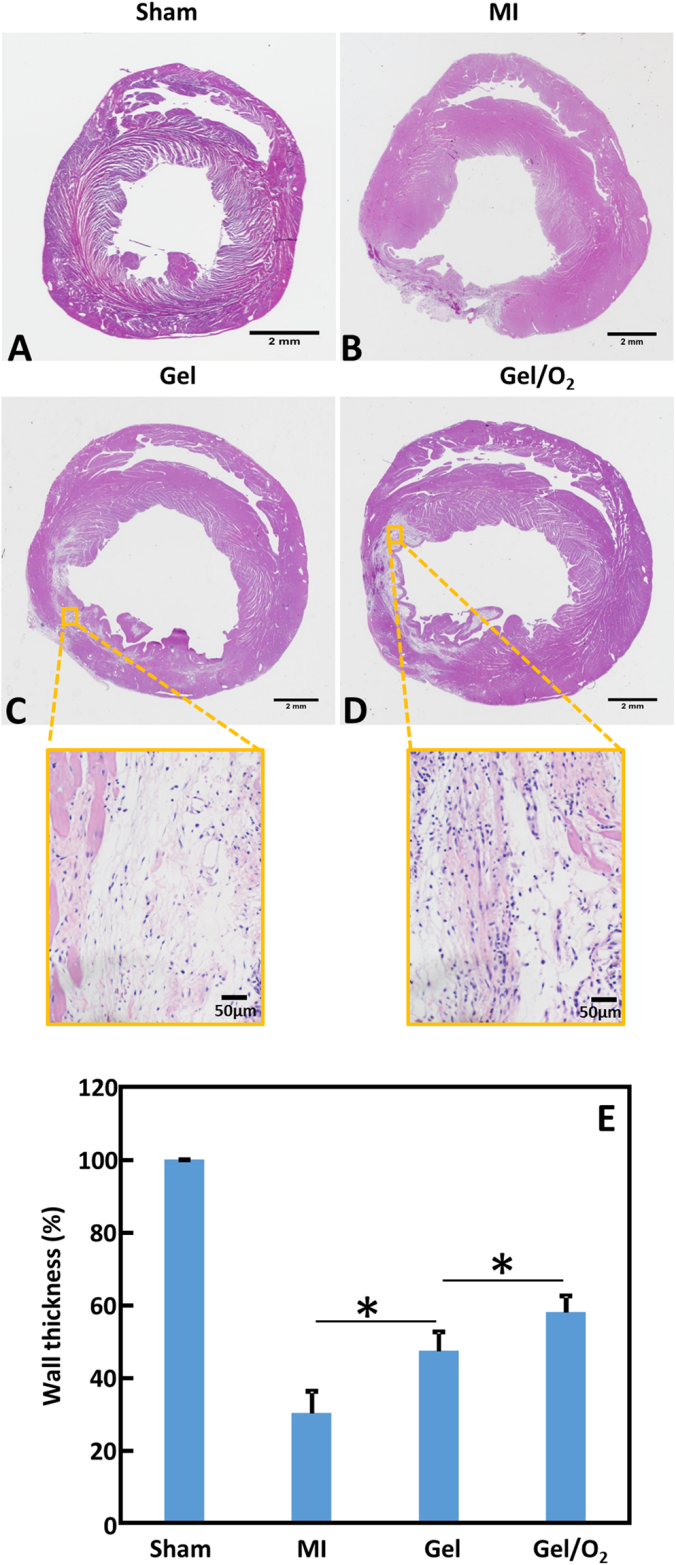
H&E staining and relative wall thickness of the infarcted hearts 4 weeks after injection. (**A**–**D**) H&E staining of Sham (**A**); MI (**B**); Gel (**C**); and Gel/O_2_ (**D**) groups. Scale bar = 2 mm for whole heart view, and scale bar = 50 µm for higher magnification view. The higher magnification H&E images indicated that cells were infiltrated into the hydrogel; and (**E**) relative wall thickness. p < 0.05.

To examine the effect of released oxygen on the remodeling of infarcted left ventricles, hydrogel encapsulated with oxygen release microspheres and catalase (Gel/O_2_ group) was injected into the infarcted rat hearts 30 min after MI. Sham, infarcted hearts without treatment (MI group), and infarcted hearts injected with hydrogel only (Gel group) were used as controls. Four weeks after injection, substantial left ventricular dilation and wall thinning were found for the MI group (Fig. 7). Injection of Gel and Gel/O_2_ groups appeared to have less dilation and wall thinning (Fig. 7). The thickness of myocardium at infarcted area was quantified from H&E images, and normalized to the left ventricle thickness of the Sham group. Figure 7 demonstrates that wall thickness of the MI group was only 30.2% of the Sham group at 4 weeks post MI. Injection of hydrogel only (Gel group) significantly increased wall thickness to 47.4% of the Sham group (p < 0.001, Gel group vs. MI group). Injection of oxygen release system further increased wall thickness to 58.0% of the Sham group (p < 0.01, Gel/O_2_ group vs. Gel group). The above results suggested that injection of hydrogel and oxygen release system attenuated adverse remodeling after MI. In addition, higher magnification H&E images of the infarcted area demonstrated that cells infiltrated into the hydrogel (Fig. 7).

### Effect of oxygen release system on host cell survival and proliferation

**Figure 8:**
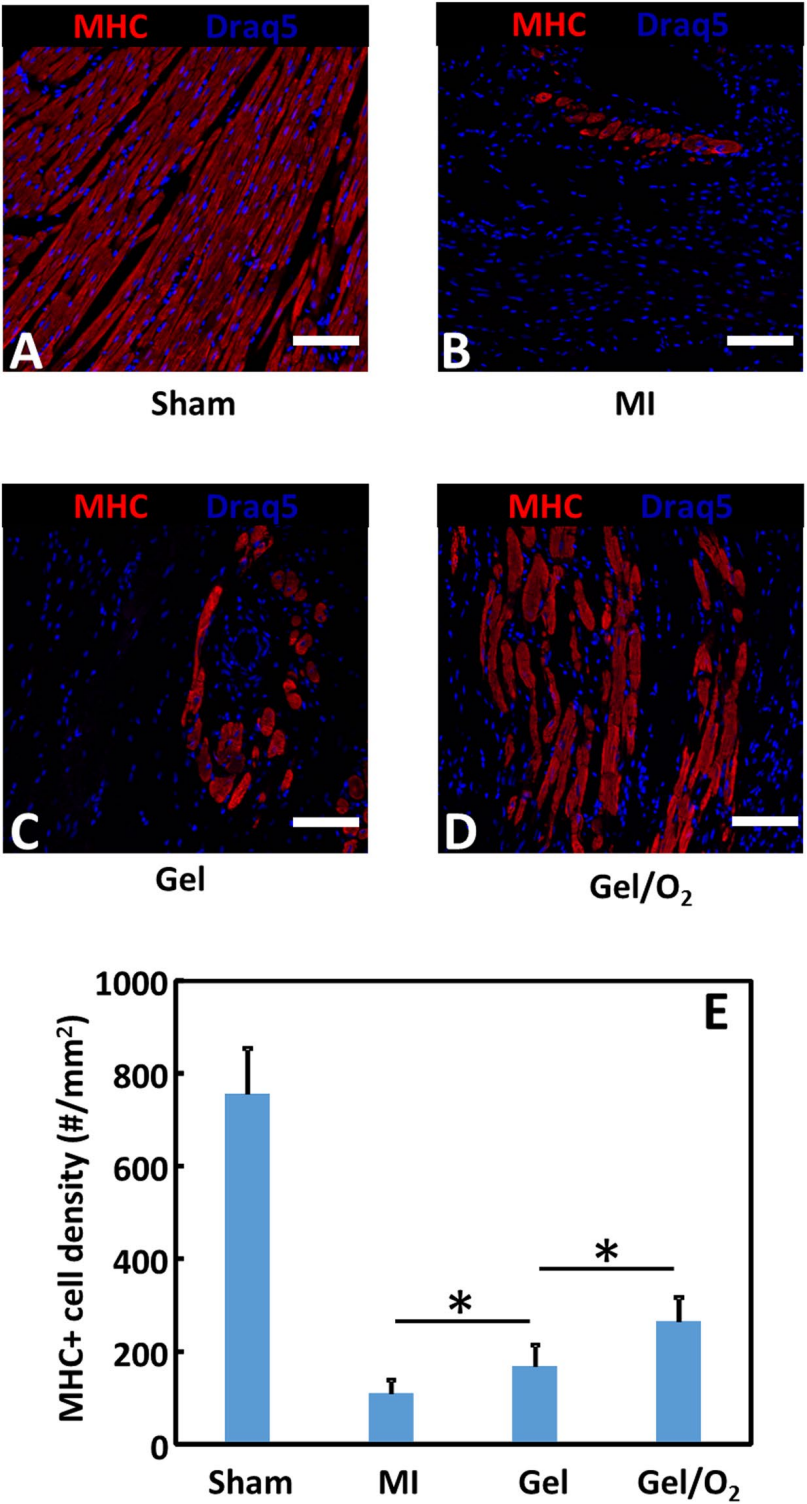
MHC staining and MHC+ cell density of the infarcted hearts 4 weeks after injection. (**A**–**D**) MHC staining of Sham (**A**); MI (**B**); Gel (**C**); and Gel/O_2_ (**D**) groups; and (**E**) MHC+ cell density in the infarcted area. Sham, MI and Gel groups were used as controls. Scale bar = 30 µm. *p < 0.05.

Cell survival was quantified for cardiomyocytes in the infarcted area at 4 weeks post MI. The surviving cardiomyocytes were identified as MHC positive cells (Fig. 8). The lowest cell density was found for the MI group where only few rounded cardiomyocytes were observed. The injection of hydrogel significantly increased cardiomyocyte density in the infarcted area (p < 0.05). Most of cells assumed round morphology with small number of cells was elongated. The injection of oxygen release system largely increased cardiomyocyte survival as the cell density was significantly greater than the Gel group (p < 0.01, Gel/O_2_ group vs. Gel group). Interestingly, most of the surviving cardiomyocytes were elongated with similar morphology as those in the Sham group.

**Figure 9:**
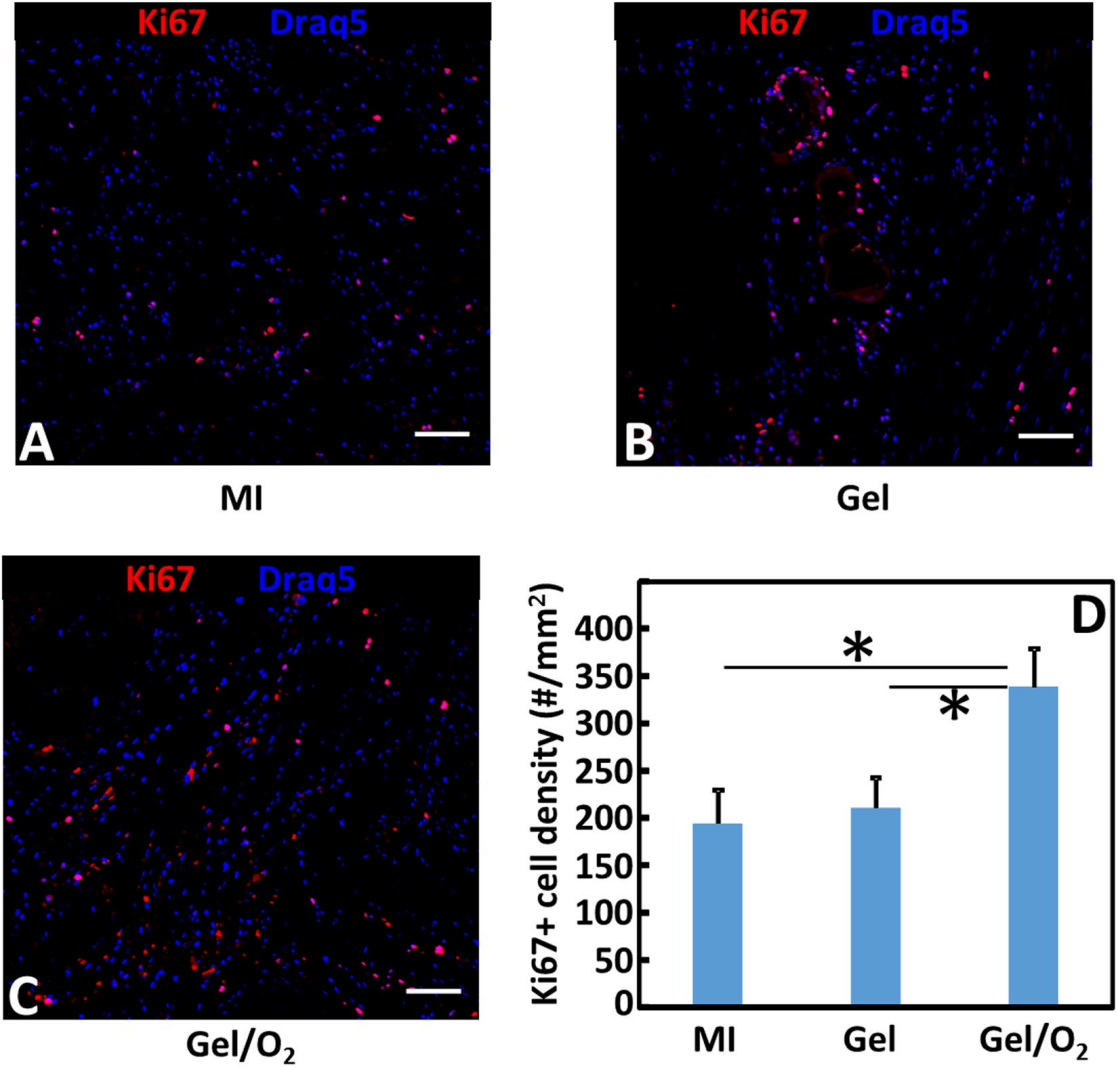
Ki67 staining of the infarcted hearts harvested 4 weeks after injection. (**A**–**C**) Ki67 staining of MI (**A**); Gel (**B**); and Gel/O_2_ (**C**) groups; and (**D**) Ki67+ cell density in the infarcted area. Scale bar = 30 µm. *p < 0.05.

To quantify host cell proliferation, Ki67 staining on the infarcted area was performed and Ki67+ cells were quantified (Fig. 9). After 4 weeks of injection, the MI group showed the minimum cell proliferation. Injection of hydrogel substantially increased cell proliferation (p > 0.05, Gel group vs. MI group). The most significant cell proliferation was observed for the Gel/O_2_ group that had oxygen release (p < 0.01, Gel/O_2_ group vs. Gel group).

### Effect of oxygen release system on cardiac fibrosis

**Figure 10:**
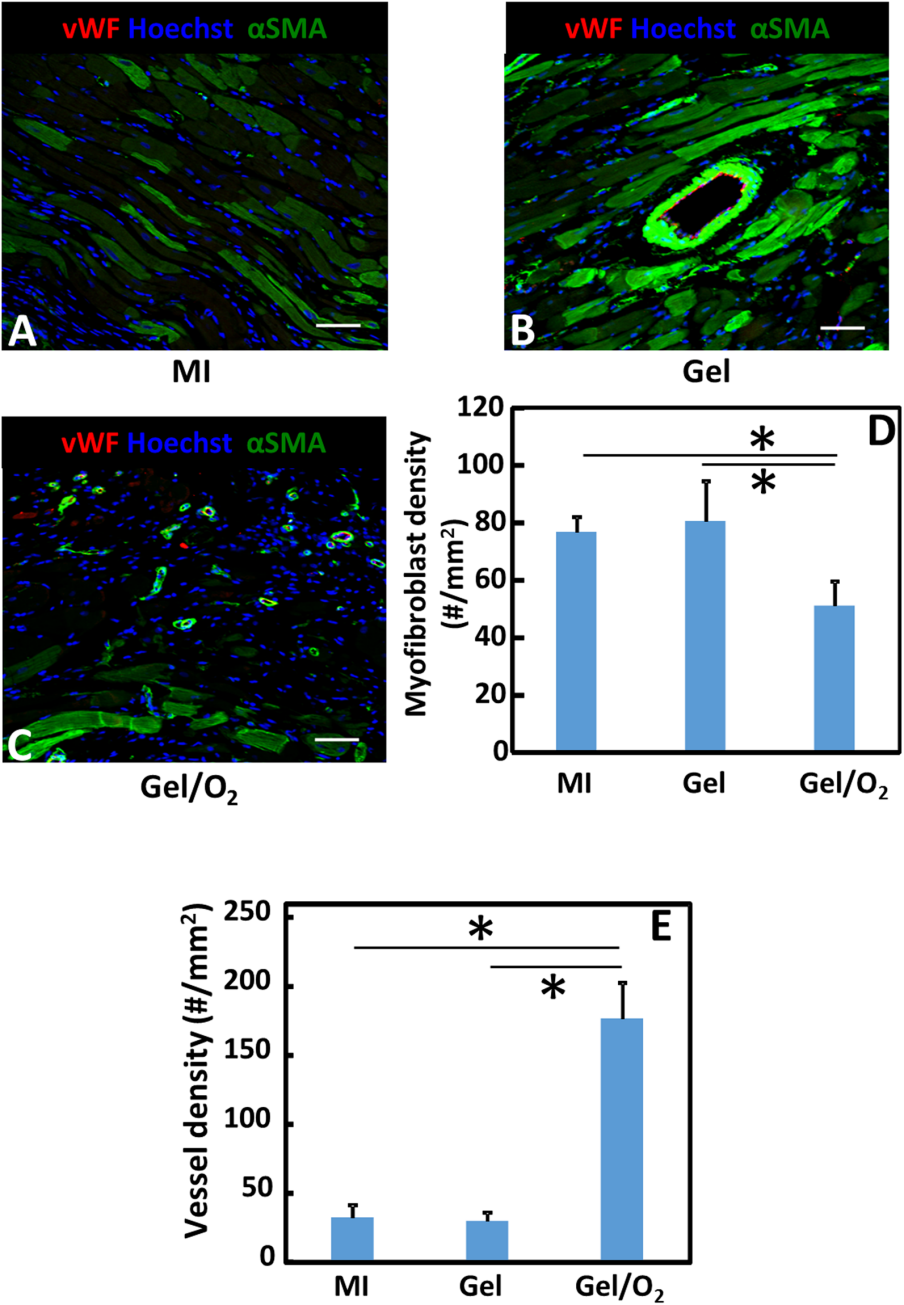
Immunohistological analysis of the infarcted region and myofibroblast density after 4 weeks of injection. (**A**–**C**) Immunohistological staining of αSMA (green), vWF (red), and Hoechst (blue) for MI (**A**); Gel (**B**); and Gel/O_2_ (**C**) groups; (**D**) myofibroblast density quantified from immunohistological images. and (**E**) Blood vessel density in the infarcted area. Scale bar = 60 μm. *p < 0.05.

**Figure 11:**
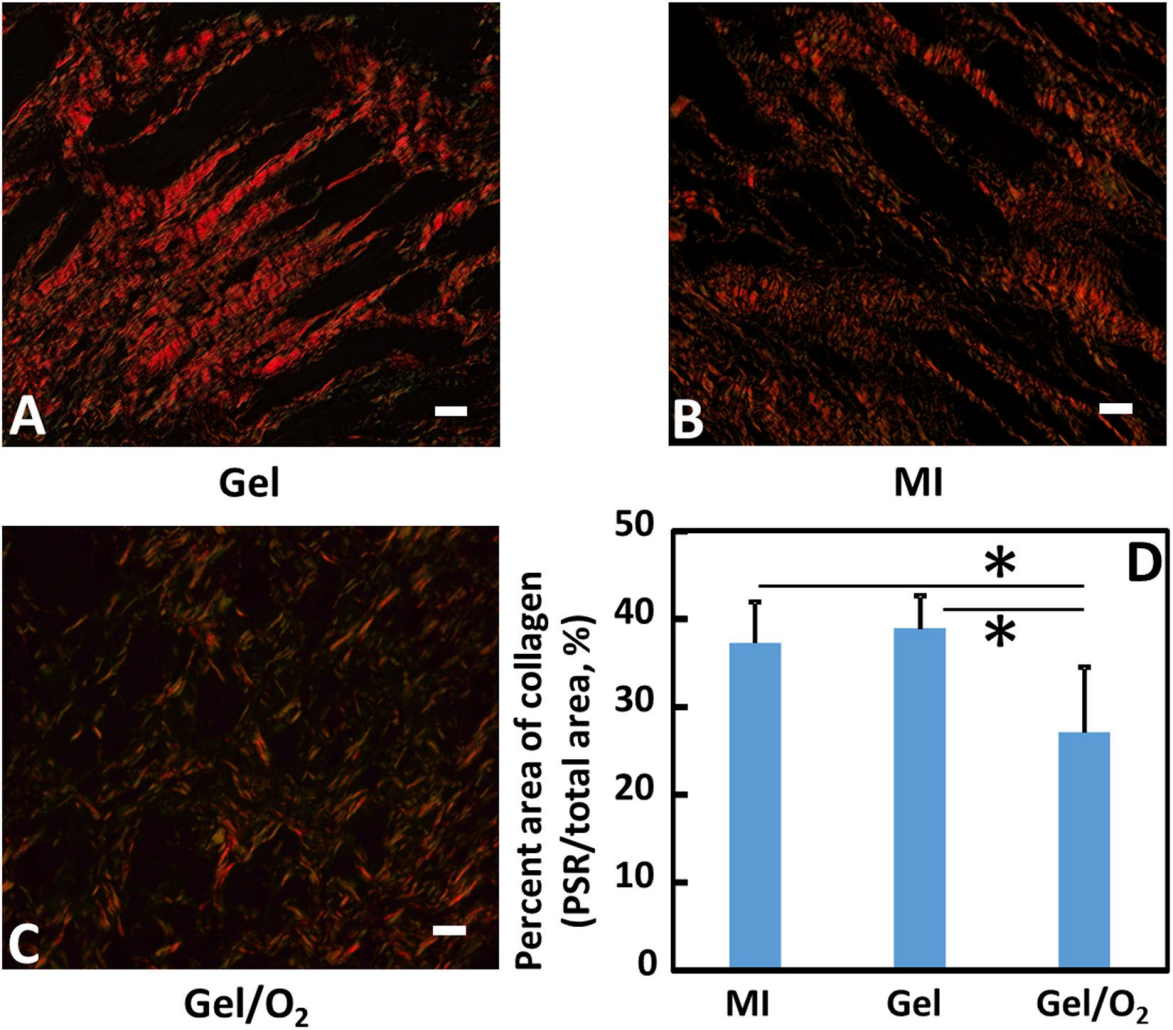
Picrosirius red staining of the infarcted hearts harvested 4 weeks after injection. Views were taken at the infarcted region of the MI (**A**); Gel (**B**); and Gel/O_2_ (**C**) groups. Total area of collagen (**D**) was analyzed from the images. Scale bar = 20 µm. *p < 0.05.

Cardiac fibrosis is characterized by increased myofibroblast density and collagen content in the infarcted tissue. Myofibroblasts are mainly responsible for collagen secretion. To determine the efficacy of oxygen release on myofibroblast formation, infarcted tissue was stained for αSMA and vWF (Fig. 10). Myofibroblasts are αSMA+ cells that are not co-localized with vWF+ endothelial cells in the vessels. Figure 10 demonstrates that myofibroblast density was significantly lower in the Gel/O_2_ group than in the MI and Gel groups (p < 0.01, Gel/O_2_ group vs. Gel group or MI group). The injection of hydrogel did not affect myofibroblast formation compared to MI group (p > 0.1). Picrosirius red staining was utilized to identify collagen in the infarcted area (Fig. 11). Total collagen content in the MI and Gel groups was significantly higher than that in the Gel/O_2_ group (p < 0.05, Gel/O_2_ group vs. MI and Gel groups). There was no significant difference between MI and Gel groups (p > 0.05). The above results demonstrated that oxygen release attenuated cardiac fibrosis.

### Effect of oxygen release system on tissue inflammatory and ROS content in infarcted hearts

**Figure 12:**
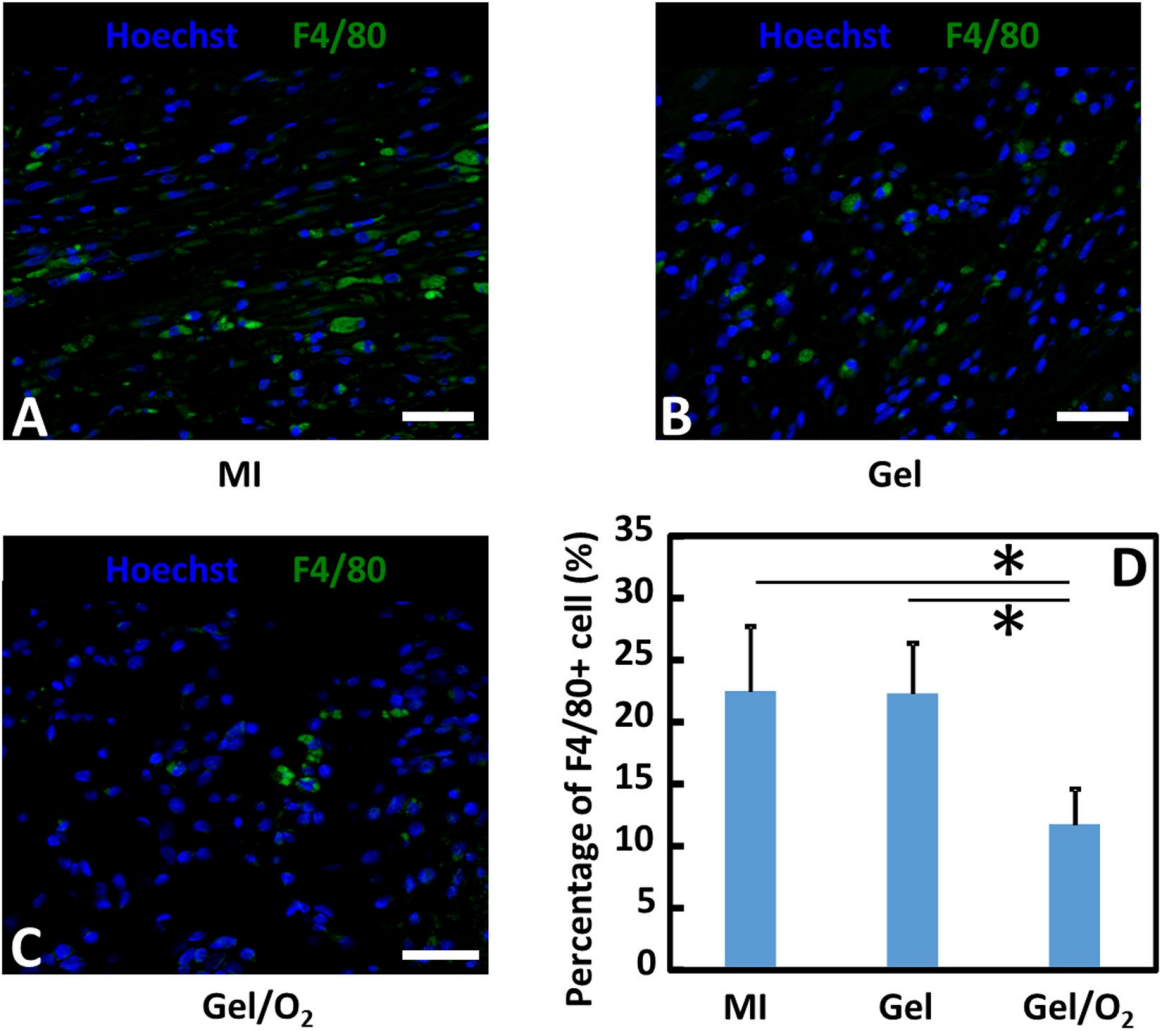
F4/80 staining and F4/80+ cell density in the infarcted area 4 weeks after injection. (**A**–**C**) F4/80 staining for MI (**A**); Gel (**B**); and Gel/O_2_ (**C**) groups; and (**D**) F4/80+ cell density quantified from images. Scale bar = 30 μm. *p < 0.05.

Tissue inflammation in the infarcted area was characterized at 4 weeks post MI by F4/80 positive macrophages (Fig. 12). The percentage of F4/80 positive cells was similar in MI group and Gel group. Injection of oxygen release system into the infarcted hearts significantly decreased percentage of F4/80 positive cells compared to MI and Gel groups (p < 0.01, Gel/O_2_ group vs. MI and Gel groups).

**Figure 13:**
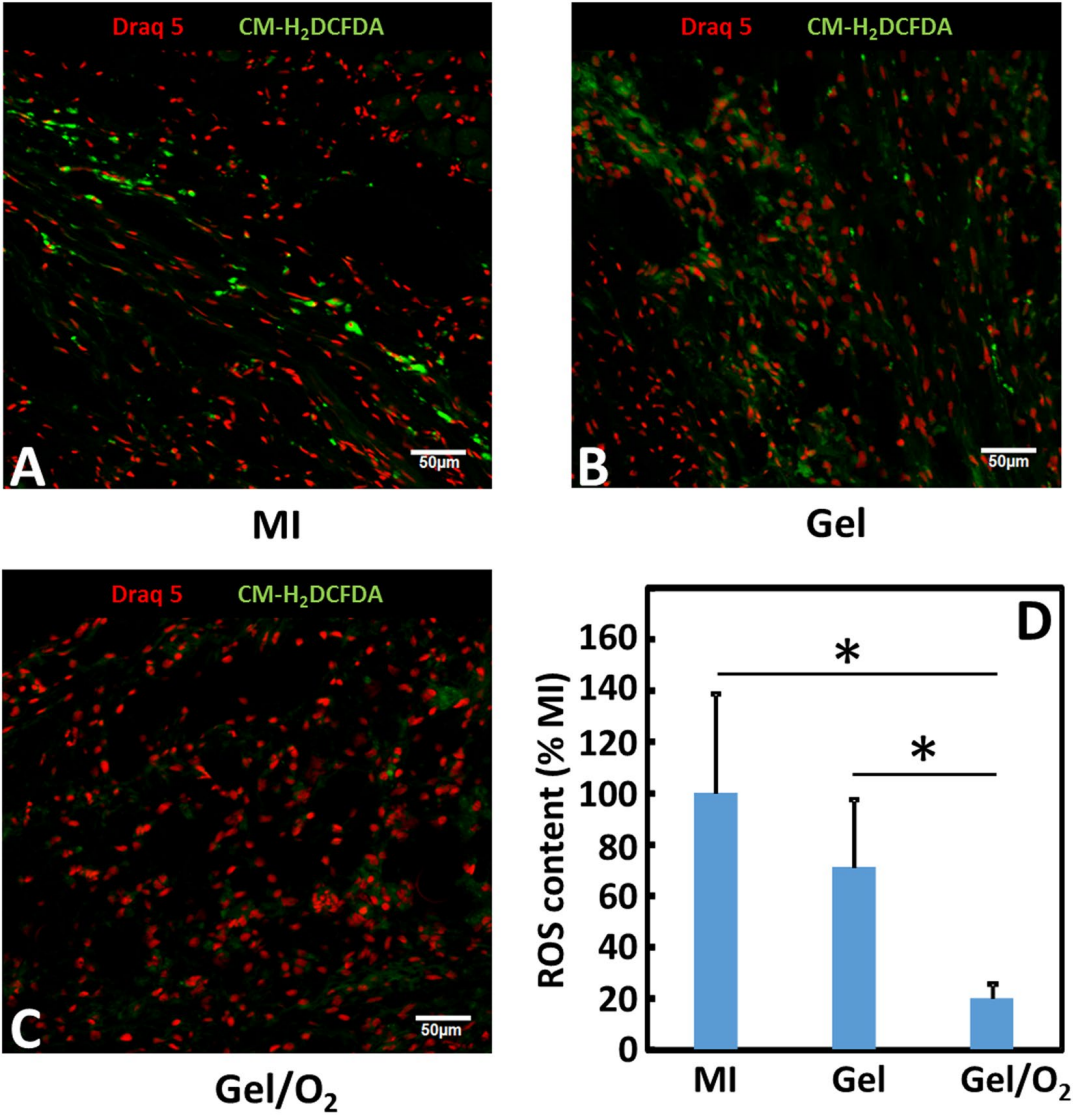
ROS staining of the infarcted hearts harvested 4 weeks after injection. (**A**–**C**) CM-H2DCFDA staining for MI (**A**); Gel (**B**); and Gel/O_2_ (**C**) groups; and (**D**) ROS content quantified from images. Scale bar = 50 μm. *p < 0.05.

To determine whether oxygen release in the infarcted hearts increased ROS content in the tissue, ROS staining was performed in the MI, Gel, and Gel/O_2_ groups (Fig. 13). The mean gray value of each cell in each surgery group was measured to evaluate the total ROS content in each group. MI group exhibited the highest ROS content. Injection of hydrogel slightly decreased ROS content (p > 0.5, Gel group vs. MI group). Interestingly, the injection of oxygen release system did not increase but significantly decreased ROS content (p < 0.01). The above results demonstrated that oxygen release in the infarcted heart tissue significantly attenuated tissue inflammation and ROS formation.

### Effect of oxygen release on infarcted tissue angiogenesis and cardiac function

**Figure 14:**
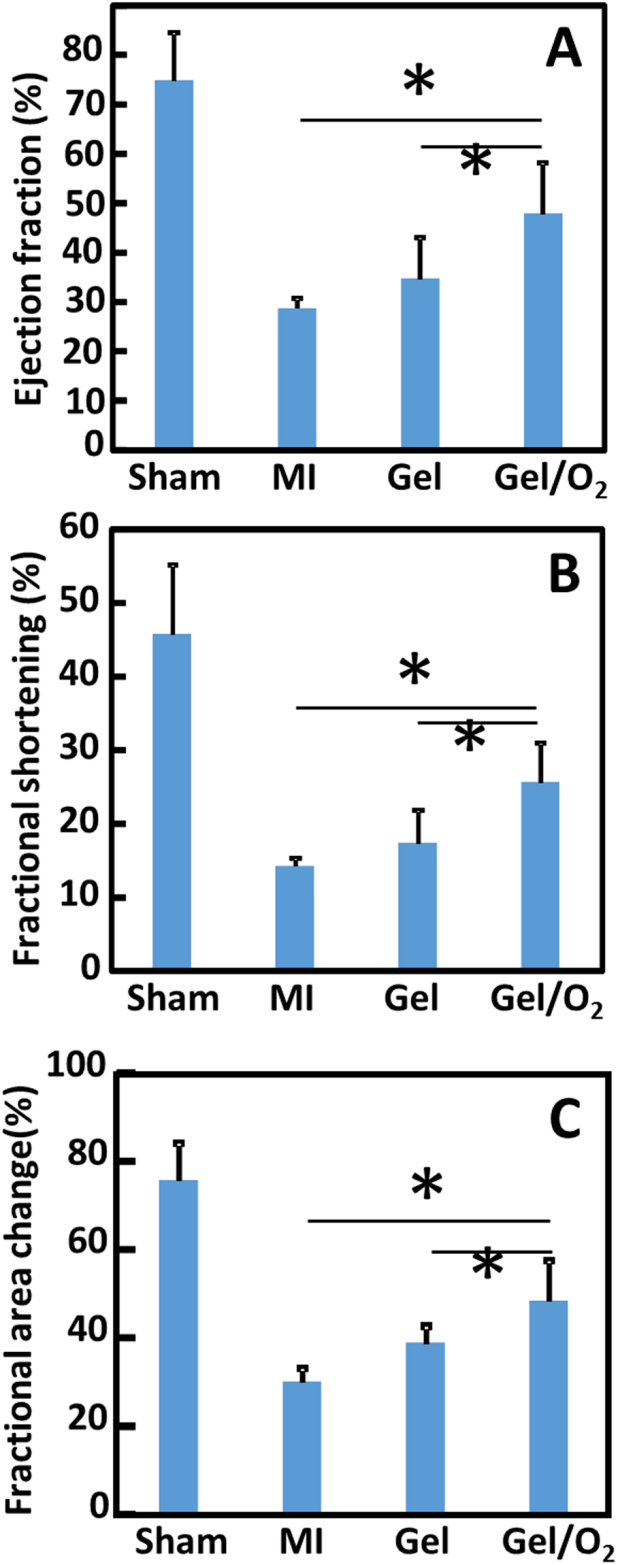
Echocardiographic analysis of Sham, MI, Gel, and Gel/O_2_ groups. (**A**) Ejection fraction; (**B**) Fractional shortening; (**C**) Fractional area change. *p < 0.05.

To investigate if oxygen release can increase infarcted tissue angiogenesis, vessel density was quantified for the MI, Gel, and Gel/O_2_ groups (Fig. 10E). The MI and Gel group had similar vessel density (p > 0.1). The injection of oxygen release system remarkably increased vessel density compared to MI and Gel groups (p < 0.01, Gel/O_2_ group vs. Gel and MI groups). Cardiac function was characterized by echocardiography. Ejection fraction (EF), fractional shortening (FS), and fractional area change (FAC) were quantified (Fig. 14) 4 weeks of injection, the Gel group exhibited substantially greater EF, FS, and FAC than the MI group (p > 0.05). Compared to the Gel group, the Gel/O_2_ group showed significantly higher EF, FS, and FAC (p < 0.05 for EF, FS and FAC). These results suggested that oxygen release promoted tissue angiogenesis and increased cardiac function.

## Discussion

In this work, an oxygen release system was developed to rescue cardiac cells and promote cardiac repair after acute MI. The system was able to sustained release oxygen to continuously oxygenate the metabolic-demanding cardiac cells and augment their survival. Controlled oxygen release to heart tissue cannot be achieved by current oxygen delivery approaches like hyperbaric oxygen therapy where tissue oxygen content decreases shortly after the treatment is finished. The oxygen delivery system was specifically injected into the infarcted tissue. The localized oxygen delivery is advantageous over systemic oxygen delivery as higher tissue oxygen content may be achieved. Systemic oxygen delivery relies on blood flow to transport oxygen to the infarcted hearts. Yet the damaged heart tissue is poorly vascularized.

The oxygen release system was based on a thermosensitive and fast gelation PNIPAAm hydrogel, and oxygen release microspheres. The hydrogel was served as carrier for the oxygen release microspheres. PNIPAAm based hydrogels have good biocompatibility and can solidify at 37 °C without using crosslinkers^55,56^. The hydrogel solution in this work was able to solidify within 7 s at 37 °C. This is beneficial for myocardial injection as the hydrogel may quickly immobilize in the heart tissue, thus increasing the retention of encapsulated microspheres in the tissue. This is evidenced in the *in vivo* study where hydrogel leaking was not observed during myocardial injection. The hydrogel solution had a thermal transition temperature of 26.7 °C and can be readily injected through 28-gauge needle. The hydrogel was degradable. The degradation product had gelation temperature of 41.2 °C. It can thus dissolve in the body fluid and be removed from body. These properties allow the developed hydrogel to be suitable for cardiac therapy^57^.

The oxygen release microspheres assumed core-shell structure with PVP/H_2_O_2_ as core and PLGA as shell (Fig. 2). The mechanism of oxygen release is that the PVP/H_2_O_2_ complex diffuses through the PLGA shell, and the H_2_O_2_ is converted into molecular oxygen by catalase in the hydrogel (Fig. 1). The oxygen was able to continuously release from the hydrogel for 4 weeks (Fig. 2). After 1 day of release, the oxygen level was reached 5%. The peak release was observed on day 7 where the oxygen level was 24.8%. Oxygen release may be faster *in vivo* as the enzymes in the infarcted hearts may accelerate the degradation of hydrogel and PLGA. However, it is possible that oxygen release can last for 4 weeks since the hydrogel was observed after 4 weeks of implantation.

This oxygen release system has advantages over those MgO_2_^58^, CaO_2_^59,60^, H_2_O_2_ (without forming complex)^61,62^, and fluorinated compounds^63–65^ based systems. First, the duration of release is longer. Oxygen release in the above mentioned systems typically lasted for 2 weeks or shorter. In contrast, oxygen release in our system persisted for 4 weeks. Second, our oxygen release system is safer as the released H_2_O_2_ can be quickly converted into oxygen by catalase. Therefore no free H_2_O_2_ is released from the system. Free H_2_O_2_ may be toxic to cells. The MgO_2_^58^, CaO_2_^59,60^, and H_2_O_2_ (without forming complex)^61,62^ based oxygen release systems release free H_2_O_2_, and relay only on H_2_O_2_ decomposition to form oxygen. Third, our system is more suitable for cardiac therapy than the MgO_2_ and CaO_2_-based oxygen release systems since it does not generate side product Mg^2+^ or Ca^2+^, which may lead to an abnormal ion transient in the heart tissue^66^.

Various studies have shown that a relatively low oxygen condition (5–20%) may not affect cell survival^67–71^, however, the extremely low oxygen condition (<1%) in infarcted hearts leads to extensive cell death^9,72–77^. Salvation of major cardiac cells like cardiomyocytes, endothelial cells and cardiac fibroblasts is critical for cardiac repair after MI^1–5^. To investigate the effect of oxygen release on cardiac cell survival under low oxygen condition mimicking that of the infarcted hearts, cells were seeded into a 3D collagen model and cultured under 1% oxygen condition. This oxygen concentration has been commonly used to simulate the pathological hypoxic condition in infarcted hearts^78,79^. Cardiomyocytes, cardiac fibroblasts and endothelial cells experienced significant death under 1% oxygen condition after 2 days of culture (Fig. 3). The injection of oxygen release system significantly augmented the survival of all 3 cell types (Fig. 3). The improved cell survival is likely due to cell oxygen content increase. One of the limitations of 3D collagen model is that cell survival can only be evaluated for 2 days due to substantial shrinkage.

Consistent with *in vitro* results, injection of the oxygen release system into infarcted hearts increased cardiomyocyte survival. Figure 8 demonstrates that the number of MHC positive cardiomyocytes was significantly greater in the group with oxygen release (Gel/O_2_ group) than that without oxygen release (Gel group). In addition, the survived cardiomyocytes in the oxygen release group showed similar morphology as the healthy cardiomyocytes in the Sham group (Fig. 8). Oxygen release also promoted cell proliferation in the infarcted region (Fig. 9). Ki67 staining results demonstrated that the density of Ki67 positive cells in the Gel/O_2_ group was significantly higher than in Gel group. Since adult cardiomyocytes do not proliferate, the Ki67 positive cells likely contain endothelial cells and cardiac fibroblasts. This is indirectly evidenced by greater vessel density in the Gel/O_2_ group than in the Gel group (Fig. 14).

One of the concerns when using oxygen therapy is ROS generation as high oxygen concentration may increase ROS content in cells and tissues, leading to cell death. Within the statistical power of the *in vitro* study in Fig. 4, ROS content of cardiomyocytes, cardiac fibroblasts, and endothelial cells in the oxygen release group did not significantly increase compared to that in the normal oxygen condition. Interestingly, the released oxygen significantly decreased ROS content in the infarcted hearts after 4 weeks of implantation (Fig. 13). These *in vitro* and *in vivo* results demonstrated that the released oxygen concentration was appropriate for myocardial repair. The reduced ROS content in the infarcted tissue is possibly resulted from decreased macrophage density (Fig. 12). Macrophages are main source of ROS after MI^80^.

Decrease of myofibroblast formation is critical to reduce cardiac fibrosis. After MI, TGFβ expression is upregulated. This promotes myofibroblast formation primarily through TGFβ binding to TGFβRIIs on cardiac fibroblasts^81^. In the infarcted hearts, cardiac fibroblast is one of the major sources of TGFβ^81^. We found that the extremely low oxygen condition (1% O_2_) significantly upregulated the expressions of TGFβ and TGFβRII on cardiac fibroblasts. However, oxygen release significantly downregulated both expressions (Fig. 5). This reduces the possibility of TGFβ binding to TGFβRII of cardiac fibroblasts resulting in decreased myofibroblast density. It is confirmed by *in vitro* cardiac fibroblast differentiation study where oxygen release significantly decreased αSMA, CTGF and collagen1A1 expressions at mRNA level, and αSMA expression at protein level (Fig. 6). After injecting into infarcted hearts for 4 weeks, the oxygen release group (Gel/O_2_ group) showed significantly lower myofibroblast density and total collagen content than the Gel group that had no oxygen release (Figs 10 and 11). This is consistent with *in vitro* findings. These results demonstrated that oxygen release significantly decreased myofibroblast formation and attenuated cardiac fibrosis after MI.

To examine the effect of oxygen release on left ventricular geometry after MI, the oxygen release system (Gel/O_2_ group) was injected into the infarcted area. MI and Gel groups were used as controls. Four weeks after injection, wall thickness of the Gel group was significantly greater than that of the MI group (Fig. 7). This is due to bulking effect of the hydrogel^82^. Similar effect was reported in other studies using different types of hydrogels^83,84^. The injection of Gel/O_2_ group further increased wall thickness (Fig. 7). This increase is likely the contribution of hydrogel as well as cardiac cells saved by the released oxygen. Figures 8 and 9 demonstrate that densities of the surviving cardiomyocytes and proliferating cells were significantly higher in the Gel/O_2_ group. It is also possible that the enhanced angiogenesis contributed to the wall thickness increase as the Gel/O_2_ group had significantly greater vessel density than the Gel group (Fig. 10E). The released oxygen may promote endothelial cell survival and proliferation under ischemic conditions of the infarcted hearts, resulting in the enhanced angiogenesis.

Cardiac function was examined 4 weeks post-MI. Injection of hydrogel only (Gel group) slightly increased EF, FS and FAC compared to the MI group. This is likely attributed to the wall stress decrease after hydrogel injection. Previous studies have shown that applying external matrix with suitable mechanical properties can effectively reduce the evaluated wall stress after MI^84^. The injection of oxygen release system significantly improved cardiac function (EF, FS, and FAC) compared to the Gel group (Fig. 14). The increase in cardiac function in the oxygen release group is possibly resulted from the mechanical support of hydrogel, increased cell survival and proliferation, greater angiogenesis, reduced inflammation, and decreased cardiac fibrosis. Overall, the above results demonstrated the benefits of using controlled oxygen release to protect cardiac cells and promote myocardial repair after MI.

The developed oxygen release system may be delivered into the infarcted hearts during coronary revascularization after MI^85^. Acute MI model was used in this work to evaluate the efficacy of oxygen release in promoting cardiac cell survival and cardiac repair. The major limitation of this model is its relatively low clinical relevance since patients may not be able to receive this type of treatment shortly after MI. However, this animal model has been widely used to investigate the efficacy of cell and biomaterial therapies after MI^84–88^.

## Methods

### Materials

All materials were purchased from Sigma-Aldrich unless otherwise stated. N-isopropylacrylamide (NIPAAm, TCI) was recrystallized in hexane before polymerization. 2-hydroxyethyl methacrylate (HEMA, TCI) was used after removing inhibitors through a column filled with inhibitor remover. Poly(lactide-co-glycolic acid) (PLGA) with LA/GA ratio of 50/50 ratio and inherent viscosity of 0.55–0.75 dL/g was purchased from Lactel. Hydrogen peroxide (30% aqueous solution), poly(N-vinylpyrrolidone) (PVP, Fisher Scientific) with molecular weight of 40–90 kDa, and bovine liver catalase (2000–5000 units/mg) were used as received.

### Synthesis and characterization of hydrogel

The hydrogel was synthesized by free radical polymerization of NIPAAm, HEMA, and a macromer acrylate-oligolactide (AOLA). In brief, NIPAAm, HEMA, and AOLA with molar ratio of 86/10/4 were dissolved in 150 mL 1,4-dioxane under the protection of nitrogen. Initiator benzoyl peroxide was then added. The polymerization reaction was conducted at 70 °C for 24 h with continuous stirring. The solution was precipitated in hexane. The obtained polymer was purified twice using THF as dissolving solvent and diethyl ether as precipitation solvent^82^.

Hydrogel solution (20 wt%) was prepared by dissolving the synthesized polymer in Dulbecco’s modified phosphate buffer saline (DPBS) at 4 °C. The thermal transition temperature of the solution was measured utilizing DSC over a temperature range of 0 °C and 60 °C with a heating rate of 10 °C/min^9,89^. Injectability of the hydrogel solution was tested by injecting the solution through a 28-gauge needle typically used for tissue injection^90^. Gelation time of the hydrogel solution was determined using a temperature controllable Olympus 1 × 71 microscope^9^. The time needed for the 4 °C hydrogel solution to become completely opaque at 37 °C was considered as gelation time. Hydrogel degradation was conducted in DPBS at 37 °C for 4 weeks. Weight loss was determined. At least 5 samples were used for degradation study.

### Fabrication of oxygen release microspheres

The oxygen release microspheres were fabricated using co-axial elctrospraying technique (Fig. 1). The use of co-axial device allows to fabricate microspheres with core-shell structure^9^. The inner stream was PVP/H_2_O_2_ complex, and the outer steam was PLGA solution (5 wt% in dichloromethane). The molar ratio of H_2_O_2_ and repeating unit of vinylpyrrolidone was 4.5. This ratio allowed the complex to have enough flowability for electrospraying. During the fabrication, the co-axial device was charged at a voltage of +15 kV. The collector aluminum pan was charged at a voltage of −10 kV. The flow rates for the H_2_O_2_/PVP complex and PLGA were 0.2 and 1 mL/h, respectively. After fabrication, the microspheres were freeze-dried for 2 h and stored at −20 °C.

### Characterization of oxygen release kinetics

Oxygen release from the microspheres was tested in DPBS supplemented with 1 mg/mL of catalase under 1% O_2_ condition. To determine oxygen level, an oxygen-sensitive dye luminophore Ru(Ph_2_phen_3_)Cl_2_ was used. Besides, an oxygen-insensitive flurophore (rhodamine-B) was utilized as a reference. These two dyes were entrapped in a polydimethylsiloxane (PDMS) membrane. The advantage of using PDMS is that it allows oxygen to interact with dyes due to its high oxygen permeability, while preventing water to penetrate inside owing to its high hydrophobicity. The PDMS membrane was punched into disks with 6 mm in diameter, and placed into a 96-well plate. Two hundred microliters of DPBS supplemented with 1 mg/mL bovine catalase was then added to each well. The plate was placed in a hypoxic incubator (1% O_2_) to balance the oxygen level in the DPBS. After 24 h, 50 mg of microspheres were quickly added to each well under 1% O_2_ environment, and the plate was sealed. The release kinetics was tested in the 1% O_2_ incubator at 37 °C (n = 5 for each group). Oxygen release was measured for 4 weeks. At each time point, the fluorescence intensities were measured at 610 nm for Ru(Ph_2_phen_3_)Cl_2_ and 576 nm for rhodamine-B (excited at 543 nm) by a fluorescent plate reader. The oxygen level was determined according to calibration curve.

### Culture of cardiac fibroblasts, cardiomyocytes and endothelial cells

Rat cardiac fibroblasts (Cell Applications, San Diego, CA) were cultured using DMEM supplemented with 10% FBS and 1% penicillin-streptomycin. The cells were incubated under 21% O_2_, 5% CO_2_, and 37 °C conditions. Human umbilical vein endothelial cells (HUVECs) were purchased from Lonza (Walkersville, MD). EGM growth medium supplemented with Endothelial Cell Growth Kit (ATCC, Manassas, VA) was used for culture. The cells between passages 4–7 were used for the studies. Rat neonatal cardiomyocytes (Lonza) were cultured in high-glucose DMEM medium supplemented with 10% FBS and 1% penicillin-streptomycin.

### Characterization of the effect of oxygen release on cardiac fibroblast, cardiomyocyte and endothelial cell survival under hypoxic conditions

To deliver oxygen release microspheres, they were encapsulated into the above synthesized hydrogel. The hydrogel solution (4 wt%) was mixed with bovine catalase (1 mg/mL) followed by sterilization under UV light for 30 min. The freeze-dried oxygen release microspheres were sterilized separately under UV light. The hydrogel solution and the microspheres (50 mg/mL) were mixed right before injection into the 3D collagen model encapsulated with cells to investigate the effect of oxygen release on cell survival under hypoxic conditions.

A 3D collagen model was used to mimic the scenario when the oxygen release system is injected into myocardium after MI. In brief, bovine type I collagen was dissolved in acetic acid solution. The formed collagen solution was then mixed with DMEM supplemented with 10% FBS, and 2 million/mL cells. The mixture was finally neutralized by 0.1 M NaOH^91^. The final collagen concentration was 2 mg/mL. The neutralized mixture was added into 48-well plates (500 µL/well). The plates were placed in a 37 °C incubator for gelation for 45 min to form a construct. The oxygen release system (hydrogel loaded with 50 mg/mL oxygen release microspheres, and 1 mg/mL catalase), or hydrogel alone was then injected into constructs with 50 µL/injection. A total of 5 injections were performed relatively uniformly in each construct. The constructs were cultured in an incubator with 1% O_2_. The constructs cultured under normal oxygen condition were used as control.

Cell survival was characterized after 48 h of culture. At least 4 constructs were collected for each experimental group, and digested by papain solution at 60 °C for 24 h. The double-stranded DNA (dsDNA, for live cells) content was measured by PicoGreen assay (Invitrogen)^9^. The dsDNA content under hypoxia condition was normalized to that in the normal condition. To image live cells, they were labeled with a live cell tracker CMFDA (5-chloromethylfluorescein diacetate) before seeding into collagen constructs^92^. After 48 h of culture. the cells were imaged using a confocal microscope (Olympus FV1000). At least 3 constructs were imaged for each experimental group.

Cell ROS content was characterized to determine whether oxygen release increased cell oxidative stress that may cause cell death. In brief, cells were trypsinized from the culture flask after CM-H_2_DCFDA (5 µM in PBS) staining for 30 min. The cells were then seeded into the collagen gel using the method described above. After 48 h of culture under 1% O_2_ condition or normal condition, samples were harvested, and the cells were imaged by a confocal microscope (Olympus FV1000). Z-stack images were taken in the 30 µm thickness. Four constructs were used for each experimental group. At least 4 different areas were imaged for each construct. All of the images were analyzed by Image J software, the mean gray value in each image was obtained for quantification^92^.

### Characterization of the effect of oxygen release on cardiac fibroblast differentiation into myofibroblast under hypoxic conditions

To investigate whether oxygen release had impact on cardiac fibroblast differentiation into myofibroblast, the oxygen release system was injected into the 3D collagen model described above. This model mimics the *in vivo* scenario when the oxygen release system is injected into infarcted heart tissue^91^. In addition, the collagen itself in this model does not change cardiac fibroblast phenotype for at least 48 h^91^. Cardiac fibroblasts were seeded into the collagen gel at the final cell density of 2 million/mL. The constructs were cultured in the culture medium with the addition of TGFβ1 (5 ng/mL) under 1% O_2_ condition.

The effect of oxygen release on cardiac fibroblast phenotype was characterized at the mRNA level by real time RT-PCR, and at the protein level by immunohistochemical analysis. RNA of the samples was isolated using TRIzol^93–95^. cDNA was synthesized at 1 µg/reaction using a High Capacity cDNA Reverse Transcription kit (ABI). The primer sequence (forward and reverse), melting temperature, and expected product sizes are listed in Table 1. Real-time RT-PCR was conducted in quadruplicate for each sample using SYBR Green master mix on LightCycler® 480 System (Roche). β-Actin was used as the internal reference. Fold of increase was calculated using ΔΔCt method^93–95^.

**Table 1:** Primers used for real time RT-PCR.

Primer	Sense	Sequence	Tm(°C)
β-actin	Forward	5′-ACTCTGTGTGGATTGGTGGC-3′	60
	Reverse	5′-AGCTCAGTAACAGTCCGCCT-3′	61
αSMA	Forward	5′-CATCAGGAACCTCGAGAAGC-3′	58
	Reverse	5′-TCGGATACTTCAGGGTCAGG-3′	58
CTGF	Forward	5′-TTCACCTACAGCACGCTTGT-3′	60
	Reverse	5′-TTGGGATGGAGGGAGTTTAC-3′	57
Collagen 1A1	Forward	5′-ACTGGTATTTGCAACTGCTTTGG-3′	60
	Reverse	5′-GCGACCCACACAAGGGTCT-3′	56
TGFβ1	Forward	5′-GCGCCTGCAGAGATTCAAGTCAAC-3′	65
	Reverse	5′-GTATCAGTGGGGGTCAGCAGCC-3′	65
TGFβRII	Forward	GACCAGAAATTCCCAGCTTCT	58
	Reverse	CAACGTCTCACACACCATCTG	59

To identify myofibroblasts formed during culture, immunochemical staining of alpha smooth muscle actin (αSMA) was performed for constructs following our established protocol^82^. In brief, the constructs were fixed with 4% paraformaldehyde solution, blocked by 10% goat serum, and permeabilized with 0.1% Triton X-100. The samples were then counterstained with αSMA antibody (mouse-anti-rat) and incubated overnight at 4 °C, followed by staining with secondary antibody Alexa Fluor 546 goat-anti-rat IgG for 1 h. Hoechst 33328 was utilized to stain cell nuclei. The images were taken with a confocal microscope (Olympus FV1000). The αSMA positive myofibroblast density was quantified from at least 5 images for each group.

### Myocardial infarction and implantation of oxygen release system

All animal experiments were conducted in accordance with the National Institutes of Health Guide for handling laboratory animals and the protocol approved by the Institutional Animal Care and Use Committee of The Ohio State University. Sprague-Dawley rats (Envigo) weighted around 200 g and aged 10–12 weeks were used. MI was performed by ligation of left anterior descending coronary artery following established method in our previous report^82^. After MI, the animals were divided into 3 groups: no treatment (MI), hydrogel injection only (Gel), and oxygen release system injection (Gel/O_2_). Each group had at least 6 animals. In the treatment groups, 200 µL of hydrogel solution or oxygen release system was injected into the apical, proximal, lateral, and septal wall regions bordering the infarct, and the center of the infarct 30 min after MI with 40 µL/injection.

### Characterization of cardiac function by echocardiography

The rats were subjected to echocardiographic analysis 4 weeks after surgery following our previously established protocol^82^. Baseline was taken prior to the surgery. M-mode echocardiographic and two dimensional images were taken and analyzed using Vevo 2100 High-Resolution *in vivo* imaging system and MS400 transducer (VisualSonics, Toronto, ON, Canada). Images were acquired in a parasternal short and long axis. The dimensions of the left ventricle were measured in short axis view during diastole and systole according to the echocardiographic images.

### Histological analyses

Four weeks after injection, rat hearts were harvested. The hearts were then underwent retrograde perfusion with DPBS to rinse out the blood followed by fixing in freshly prepared 4% paraformaldehyde overnight. The sections obtained at the level of injection site were chosen for histological studies. The cardiac tissue was embedded in paraffin and sequentially sectioned along the longitudinal axis with 4 μm for each section. Sections were stained with hematoxylin and eosin (H&E), and Picrosirius red (PSR) for wall thickness and collagen composition study, respectively^96^. The thicknesses of at least 10 different areas were measured from each heart sample using ImageJ software. The relative wall thickness was defined as the ratio of myocardium thickness at infarcted area to that of normal area^97^. Type I and III collagens were determined by PSR staining images as yellowish and greenish respectively using polarized light microscopy with appropriate band-pass filters^98,99^. The percent area of collagen within the remote and MI regions was determined from images (n ≥ 10) using threshold image analysis with ImageJ software^82^.

For immunohistochemical staining, paraffin embedded cardiac tissue was sectioned at 4 µm thickness. The sections were counter stained with F4/80, Myosin Heavy Chain (MHC), Ki67, αSMA, and von willebrand factor (vWF) antibodies, respectively. Images (n ≥ 5) of the infarcted area were analyzed. Macrophages were F4/80+ cells. Its percentage was calculated as number of F4/80+ cells over total number of cells in each image. Surviving cardiomyocytes and proliferating cells in the infarcted area were identified as MHC+ and Ki67+ cells, respectively. Myofibroblasts were αSMA+ spindle shaped cells that were not co-localized with vWF+ endothelial cells. Blood vessels were recognized as vWF positive lumen. Densities of MHC+ cells, Ki67+ cells, myofibroblasts, and blood vessels were calculated as #/mm^2^.

To assess ROS content, frozen cardiac tissue was sectioned with 10 µm thickness, and the sections were stained with CM-H_2_DCFDA. Mean gray value of each cell with positive expression was measured using ImageJ software^100^.

### Statistical analysis

Statistical analysis was performed using JMP software. One way ANOVA with post-hoc Tukey-Kramer HSD test was utilized for data analysis. Data was presented as mean ± standard deviation. Statistical significance was defined as p < 0.05.

### Data availability

The datasets generated during and/or analyzed during the current study are available from the corresponding author on reasonable request.

## References

[1] Wang F (2010). Injectable, rapid gelling and highly flexible hydrogel composites as growth factor and cell carriers. Acta biomaterialia.

[2] Li Z, Guan J (2011). Hydrogels for Cardiac Tissue Engineering. Polymers.

[3] Janicki JS, Brower GL, Gardner JD, Chancey AL, Stewart JA (2004). The dynamic interaction between matrix metalloproteinase activity and adverse myocardial remodeling. Heart failure reviews.

[4] Spinale FG, Janicki JS, Zile MR (2013). Membrane-associated matrix proteolysis and heart failure. Circulation research.

[5] Tsuruda T, Costello-Boerrigter LC, Burnett JC (2004). Matrix metalloproteinases: pathways of induction by bioactive molecules. Heart failure reviews.

[6] Roy S, Khanna S, Sen CK (2004). Perceived hyperoxia: oxygen-regulated signal transduction pathways in the heart. Methods in enzymology.

[7] Roy S (2006). Transcriptome analysis of the ischemia-reperfused remodeling myocardium: temporal changes in inflammation and extracellular matrix. Physiological genomics.

[8] Li X, Tamama K, Xie X, Guan J (2016). Improving Cell Engraftment in Cardiac Stem Cell Therapy. Stem cells international.

[9] Li Z, Guo X, Guan J (2012). An oxygen release system to augment cardiac progenitor cell survival and differentiation under hypoxic condition. Biomaterials.

[10] Wang B (2015). Establishing Early Functional Perfusion and Structure in Tissue Engineered Cardiac Constructs. Critical reviews in biomedical engineering.

[11] Wang F, Guan J (2010). Cellular cardiomyoplasty and cardiac tissue engineering for myocardial therapy. Adv Drug Deliv Rev.

[12] Don CW, Murry CE (2013). Improving survival and efficacy of pluripotent stem cell-derived cardiac grafts. Journal of cellular and molecular medicine.

[13] Naumova AV (2014). Magnetic Resonance Imaging Tracking of Graft Survival in the Infarcted Heart: Iron Oxide Particles Versus Ferritin Overexpression Approach. Journal of cardiovascular pharmacology and therapeutics.

[14] Weyers JJ (2013). Effects of cell grafting on coronary remodeling after myocardial infarction. Journal of the American Heart Association.

[15] Lister Z, Rayner KJ, Suuronen EJ (2016). How Biomaterials Can Influence Various Cell Types in the Repair and Regeneration of the Heart after Myocardial Infarction. Frontiers in bioengineering and biotechnology.

[16] Pascual-Gil S, Garbayo E, Diaz-Herraez P, Prosper F, Blanco-Prieto MJ (2015). Heart regeneration after myocardial infarction using synthetic biomaterials. Journal of controlled release: official journal of the Controlled Release Society.

[17] Feric NT, Radisic M (2016). Strategies and Challenges to Myocardial Replacement Therapy. Stem cells translational medicine.

[18] Lakshmanan, R. & Maulik, N. Development of next generation cardiovascular therapeutics through bio-assisted nanotechnology. *Journal of biomedical materials research. Part B, Applied biomaterial*s, 10.1002/jbm.b.34000 (2017). 10.1002/jbm.b.34000 28950048

[19] Nadlacki B, Suuronen EJ (2016). Biomaterial strategies to improve the efficacy of bone marrow cell therapy for myocardial infarction. Expert opinion on biological therapy.

[20] Wang RM, Christman KL (2016). Decellularized myocardial matrix hydrogels: In basic research and preclinical studies. Advanced drug delivery reviews.

[21] Yanamandala M (2017). Overcoming the Roadblocks to Cardiac Cell Therapy Using Tissue Engineering. Journal of the American College of Cardiology.

[22] Zhu Y, Matsumura Y, Wagner WR (2017). Ventricular wall biomaterial injection therapy after myocardial infarction: Advances in material design, mechanistic insight and early clinical experiences. Biomaterials.

[23] Christman KL (2004). Injectable fibrin scaffold improves cell transplant survival, reduces infarct expansion, and induces neovasculature formation in ischemic myocardium. Journal of the American College of Cardiology.

[24] Kutschka I (2006). Collagen matrices enhance survival of transplanted cardiomyoblasts and contribute to functional improvement of ischemic rat hearts. Circulation.

[25] Lu S (2010). Both the transplantation of somatic cell nuclear transfer- and fertilization-derived mouse embryonic stem cells with temperature-responsive chitosan hydrogel improve myocardial performance in infarcted rat hearts. Tissue engineering. Part A.

[26] Cheng K (2012). Functional performance of human cardiosphere-derived cells delivered in an *in situ* polymerizable hyaluronan-gelatin hydrogel. Biomaterials.

[27] Shuvy M (2013). Oxygen therapy in acute coronary syndrome: are the benefits worth the risk?. European heart journal.

[28] Maroko PR, Radvany P, Braunwald E, Hale SL (1975). Reduction of infarct size by oxygen inhalation following acute coronary occlusion. Circulation.

[29] Loomba RS, Nijhawan K, Aggarwal S, Arora RR (2016). Oxygen in the Setting of Acute Myocardial Infarction: Is It Really a Breath of Fresh Air?. Journal of cardiovascular pharmacology and therapeutics.

[30] Kelly RF, Hursey TL, Parrillo JE, Schaer GL (1995). Effect of 100% oxygen administration on infarct size and left ventricular function in a canine model of myocardial infarction and reperfusion. American heart journal.

[31] Madias JE, Hood WB (1976). Reduction of precordial ST-segment elevation in patients with anterior myocardial infarction by oxygen breathing. Circulation.

[32] Rawles JM, Kenmure AC (1976). Controlled trial of oxygen in uncomplicated myocardial infarction. British medical journal.

[33] Ukholkina GB, Kostianov I, Kuchkina NV, Grendo EP (2005). & Gofman Ia, B. [Effect of oxygenotherapy used in combination with reperfusion in patients with acute myocardial infarction]. Kardiologiia.

[34] Wilson AT, Channer KS (1997). Hypoxaemia and supplemental oxygen therapy in the first 24 hours after myocardial infarction: the role of pulse oximetry. Journal of the Royal College of Physicians of London.

[35] Thom SR (2011). Hyperbaric oxygen: its mechanisms and efficacy. Plastic and reconstructive surgery.

[36] Grim PS, Gottlieb LJ, Boddie A, Batson E (1990). Hyperbaric oxygen therapy. Jama.

[37] Khan M (2012). Oxygen cycling in conjunction with stem cell transplantation induces NOS3 expression leading to attenuation of fibrosis and improved cardiac function. Cardiovascular research.

[38] Dekleva M (2004). Adjunctive effect of hyperbaric oxygen treatment after thrombolysis on left ventricular function in patients with acute myocardial infarction. American heart journal.

[39] Vlahovic A (2004). Hyperbaric oxygen treatment does not affect left ventricular chamber stiffness after myocardial infarction treated with thrombolysis. American heart journal.

[40] Stavitsky Y (1998). Hyperbaric oxygen and thrombolysis in myocardial infarction: the ‘HOT MI’ randomized multicenter study. Cardiology.

[41] Warda HM (2005). Effect of intracoronary aqueous oxygen on left ventricular remodeling after anterior wall ST-elevation acute myocardial infarction. The American journal of cardiology.

[42] O’Neill WW (2007). Acute Myocardial Infarction with Hyperoxemic Therapy (AMIHOT): a prospective, randomized trial of intracoronary hyperoxemic reperfusion after percutaneous coronary intervention. Journal of the American College of Cardiology.

[43] Stone GW (2009). Effect of supersaturated oxygen delivery on infarct size after percutaneous coronary intervention in acute myocardial infarction. Circulation. Cardiovascular interventions.

[44] Li T, Liu J, Yang C (2010). Pretreatment with hemoglobin-based oxygen carriers protect isolated rat heart from myocardial infarction. Artificial cells, blood substitutes, and immobilization biotechnology.

[45] George I (2006). A polymerized bovine hemoglobin oxygen carrier preserves regional myocardial function and reduces infarct size after acute myocardial ischemia. American journal of physiology. Heart and circulatory physiology.

[46] Huang NF (2006). A rodent model of myocardial infarction for testing the efficacy of cells and polymers for myocardial reconstruction. Nature protocols.

[47] Garcia-Ruiz JM (2017). Bloodless reperfusion with the oxygen carrier HBOC-201 in acute myocardial infarction: a novel platform for cardioprotective probes delivery. Basic research in cardiology.

[48] Gholipourmalekabadi, M., Zhao, S., Harrison, B. S., Mozafari, M. & Seifalian, A. M. Oxygen-Generating Biomaterials: A New, Viable Paradigm for Tissue Engineering? *Trends in biotechnology*, 10.1016/j.tibtech.2016.05.012 (2016). 10.1016/j.tibtech.2016.05.012 27325423

[49] Momen A (2009). Coronary blood flow responses to physiological stress in humans. American journal of physiology. Heart and circulatory physiology.

[50] McNulty PH (2005). Effects of supplemental oxygen administration on coronary blood flow in patients undergoing cardiac catheterization. American journal of physiology. Heart and circulatory physiology.

[51] Bacon JR, Demas JN (1987). Determination of oxygen concentrations by luminescence quenching of a polymer-immobilized transition-metal complex. Analytical Chemistry.

[52] Kneas KA, Xu W, Demas JN, DeGraff BA (1997). Oxygen Sensors Based on Luminescence Quenching: Interactions of Tris(4,7-diphenyl-1,10-phenanthroline)ruthenium(II) Chloride and Pyrene with Polymer Supports. Applied Spectroscopy.

[53] Acosta MA, Ymele-Leki P, Kostov YV, Leach JB (2009). Fluorescent microparticles for sensing cell microenvironment oxygen levels within 3D scaffolds. Biomaterials.

[54] Galie PA, Westfall MV, Stegemann JP (2011). Reduced serum content and increased matrix stiffness promote the cardiac myofibroblast transition in 3D collagen matrices. Cardiovascular pathology: the official journal of the Society for Cardiovascular Pathology.

[55] GhavamiNejad A, SamariKhalaj M, Aguilar LE, Park CH, Kim CS (2016). pH/NIR Light-Controlled Multidrug Release via a Mussel-Inspired Nanocomposite Hydrogel for Chemo-Photothermal Cancer Therapy. Scientific reports.

[56] Aguilar LE, GhavamiNejad A, Park CH, Kim CS (2017). On-demand drug release and hyperthermia therapy applications of thermoresponsive poly-(NIPAAm-co-HMAAm)/polyurethane core-shell nanofiber mat on non-vascular nitinol stents. Nanomedicine: nanotechnology, biology, and medicine.

[57] Ma Z, Nelson DM, Hong Y, Wagner WR (2010). Thermally Responsive Injectable Hydrogel Incorporating Methacrylate-Polylactide for Hydrolytic Lability. Biomacromolecules.

[58] Harrison BS, Eberli D, Lee SJ, Atala A, Yoo JJ (2007). Oxygen producing biomaterials for tissue regeneration. Biomaterials.

[59] Oh SH, Ward CL, Atala A, Yoo JJ, Harrison BS (2009). Oxygen generating scaffolds for enhancing engineered tissue survival. Biomaterials.

[60] Pedraza E, Coronel MM, Fraker CA, Ricordi C, Stabler CL (2012). Preventing hypoxia-induced cell death in beta cells and islets via hydrolytically activated, oxygen-generating biomaterials. Proceedings of the National Academy of Sciences of the United States of America.

[61] Ng SM, Choi JY, Han HS, Huh JS, Lim JO (2010). Novel microencapsulation of potential drugs with low molecular weight and high hydrophilicity: hydrogen peroxide as a candidate compound. International journal of pharmaceutics.

[62] Bae SE, Son JS, Park K, Han DK (2009). Fabrication of covered porous PLGA microspheres using hydrogen peroxide for controlled drug delivery and regenerative medicine. Journal of controlled release: official journal of the Controlled Release Society.

[63] Chin K, Khattak SF, Bhatia SR, Roberts SC (2008). Hydrogel-perfluorocarbon composite scaffold promotes oxygen transport to immobilized cells. Biotechnology progress.

[64] White JC, Stoppel WL, Roberts SC, Bhatia SR (2013). Addition of perfluorocarbons to alginate hydrogels significantly impacts molecular transport and fracture stress. Journal of biomedical materials research. Part A.

[65] Seifu DG, Isimjan TT, Mequanint K (2011). Tissue engineering scaffolds containing embedded fluorinated-zeolite oxygen vectors. Acta biomaterialia.

[66] Ai X, Curran JW, Shannon TR, Bers DM, Pogwizd SM (2005). Ca2+/calmodulin-dependent protein kinase modulates cardiac ryanodine receptor phosphorylation and sarcoplasmic reticulum Ca2+ leak in heart failure. Circulation research.

[67] Yew TL (2012). Scale-up of MSC under hypoxic conditions for allogeneic transplantation and enhancing bony regeneration in a rabbit calvarial defect model. Journal of orthopaedic research: official publication of the Orthopaedic Research Society.

[68] Wise JK, Alford AI, Goldstein SA, Stegemann JP (2014). Comparison of uncultured marrow mononuclear cells and culture-expanded mesenchymal stem cells in 3D collagen-chitosan microbeads for orthopedic tissue engineering. Tissue Eng Part A.

[69] Lennon DP, Edmison JM, Caplan AI (2001). Cultivation of rat marrow-derived mesenchymal stem cells in reduced oxygen tension: effects on *in vitro* and *in vivo* osteochondrogenesis. Journal of cellular physiology.

[70] Ma T, Grayson WL, Frohlich M, Vunjak-Novakovic G (2009). Hypoxia and stem cell-based engineering of mesenchymal tissues. Biotechnology progress.

[71] Das R, Jahr H, van Osch GJ, Farrell E (2010). The role of hypoxia in bone marrow-derived mesenchymal stem cells: considerations for regenerative medicine approaches. Tissue engineering. Part B, Reviews.

[72] Benoit E (2011). The role of amputation as an outcome measure in cellular therapy for critical limb ischemia: implications for clinical trial design. Journal of translational medicine.

[73] Gupta NK, Armstrong EJ, Parikh SA (2014). The current state of stem cell therapy for peripheral artery disease. Current cardiology reports.

[74] Gupta PK (2013). A double blind randomized placebo controlled phase I/II study assessing the safety and efficacy of allogeneic bone marrow derived mesenchymal stem cell in critical limb ischemia. Journal of translational medicine.

[75] Li Y (2014). Primed 3D injectable microniches enabling low-dosage cell therapy for critical limb ischemia. Proc Natl Acad Sci USA.

[76] Xu Y (2016). A prosurvival and proangiogenic stem cell delivery system to promote ischemic limb regeneration. Acta biomaterialia.

[77] Li Z, Guo X, Guan J (2012). A thermosensitive hydrogel capable of releasing bFGF for enhanced differentiation of mesenchymal stem cell into cardiomyocyte-like cells under ischemic conditions. Biomacromolecules.

[78] Zhang J (2009). Salidroside protects cardiomyocyte against hypoxia-induced death: A HIF-1α-activated and VEGF-mediated pathway. European Journal of Pharmacology.

[79] Zhu H-M, Deng L (2012). Evaluation of cardiomyocyte hypoxia injury models for the pharmacological study *in vitro*. Pharmaceutical Biology.

[80] Aoyagi, T. & Matsui, T. The Cardiomyocyte as a Source of Cytokines in Cardiac Injury. Journal of cell science & therapy 2012 (2011). 10.4172/2157-7013.s5-003 PMC3594870 23493668

[81] Fan Z, Guan J (2016). Antifibrotic therapies to control cardiac fibrosis. Biomaterials research.

[82] Fan Z (2017). Sustained Release of a Peptide-Based Matrix Metalloproteinase-2 Inhibitor to Attenuate Adverse Cardiac Remodeling and Improve Cardiac Function Following Myocardial Infarction. Biomacromolecules.

[83] Blackburn NJ (2015). Timing underpins the benefits associated with injectable collagen biomaterial therapy for the treatment of myocardial infarction. Biomaterials.

[84] Yoshizumi T (2016). Timing effect of intramyocardial hydrogel injection for positively impacting left ventricular remodeling after myocardial infarction. Biomaterials.

[85] Eckhouse SR (2014). Local hydrogel release of recombinant TIMP-3 attenuates adverse left ventricular remodeling after experimental myocardial infarction. Science translational medicine.

[86] Tous E (2011). Influence of injectable hyaluronic acid hydrogel degradation behavior on infarction-induced ventricular remodeling. Biomacromolecules.

[87] MacArthur JW (2013). Sustained release of engineered stromal cell-derived factor 1-alpha from injectable hydrogels effectively recruits endothelial progenitor cells and preserves ventricular function after myocardial infarction. Circulation.

[88] Purcell BP (2014). Injectable and bioresponsive hydrogels for on-demand matrix metalloproteinase inhibition. Nature materials.

[89] Hume PS, Anseth KS (2011). Polymerizable superoxide dismutase mimetic protects cells encapsulated in poly(ethylene glycol) hydrogels from reactive oxygen species-mediated damage. Journal of biomedical materials research. Part A.

[90] Klein AW, Elson ML (2000). The history of substances for soft tissue augmentation. Dermatologic surgery: official publication for American Society for Dermatologic Surgery [et al.].

[91] Gudur M (2012). Noninvasive, quantitative, spatiotemporal characterization of mineralization in three-dimensional collagen hydrogels using high-resolution spectral ultrasound imaging. Tissue engineering. Part C, Methods.

[92] Li Z, Wang F, Roy S, Sen CK, Guan J (2009). Injectable, highly flexible, and thermosensitive hydrogels capable of delivering superoxide dismutase. Biomacromolecules.

[93] Li Z (2016). pH-Sensitive and Thermosensitive Hydrogels as Stem-Cell Carriers for Cardiac Therapy. ACS Appl Mater Interfaces.

[94] Li Z, Guo X, Palmer AF, Das H, Guan J (2012). High-efficiency matrix modulus-induced cardiac differentiation of human mesenchymal stem cells inside a thermosensitive hydrogel. Acta biomaterialia.

[95] Xu Y (2015). Regulating myogenic differentiation of mesenchymal stem cells using thermosensitive hydrogels. Acta biomaterialia.

[96] Rich L, Whittaker P (2005). Collagen and picrosirius red staining: a polarized light assessment of fibrillar hue and spatial distribution. Braz J Morphol Sci.

[97] Deng B (2015). Delivery of alginate-chitosan hydrogel promotes endogenous repair and preserves cardiac function in rats with myocardial infarction. Journal of biomedical materials research. Part A.

[98] Junqueira LC, Bignolas G, Brentani RR (1979). Picrosirius staining plus polarization microscopy, a specific method for collagen detection in tissue sections. The Histochemical journal.

[99] Namba T (1997). Regulation of Fibrillar Collagen Gene Expression and Protein Accumulation in Volume-Overloaded Cardiac Hypertrophy. Circulation.

[100] Yancey DM (2015). Cardiomyocyte mitochondrial oxidative stress and cytoskeletal breakdown in the heart with a primary volume overload. American journal of physiology. Heart and circulatory physiology.

